# Green synthesis of 2D azine-linked covalent organic framework with antibacterial activity correlated by molecular docking study and computational calculations

**DOI:** 10.1038/s41598-025-32900-3

**Published:** 2026-01-10

**Authors:** Eman Abdelnasser, Asmaa M. Fahim, Daniel T. Oyekunle, Abd El-Motaleb Mosad Ramadan, Ahmed I. Khodair

**Affiliations:** 1 Chemistry Department, Faculty of Science, Kafrelsheikh University, El-Geish Street, P.O. Box 33516, Kafrelsheikh, Egypt; 2 Green Chemistry Department, National Research Center Dokki, P.O. Box 12622, Cairo, Egypt; 3 School of Environment and Sustainability, University of Saskatchewan, 117 Science Place, Saskatoon, SK S7N 5C8 Canada

**Keywords:** Azine-linkage, Antibacterial activity, Mesopores, Docking investigation, And Theoretical analysis, Biochemistry, Chemistry, Computational biology and bioinformatics, Drug discovery, Microbiology

## Abstract

A two-dimensional covalent organic framework (2D-COF), COFTHB, was synthesized via a Schiff base condensation of terephthaldehyde and 1,4-hydrazonmethylbenzene under green, room-temperature conditions. COFTHB exhibits a mesoporous structure (pore size = 3.68 nm), excellent chemical stability, and high thermal stability up to 629 °C. It demonstrated superior antibacterial activity against both Gram-negative (*Pseudomonas aeruginosa*, *Escherichia coli*) and Gram-positive (*Enterococcus faecalis*, *Staphylococcus aureus*) bacteria compared to hydrazonmethyl benzene and a model compound (M). Molecular docking simulations revealed the interactions of COFTHB with various proteins, while Density Functional Theory (DFT) (WB97XD/6-311G) analysis of COFTHB, HB (1,4-bis(*Z*)-hydrazonomethyl benzene), and the model compound provided insights into their electronic properties, reactivity, and resonance effects through Frontier Molecular Orbitals (FMO), Electrostatic Potential (ESP), and Molecular Electrostatic Potential (MEP) analyses. These results suggest COFTHB as a promising platform for antibacterial applications in water treatment.

**Electronic supplementary material:**

The online version of this article (10.1038/s41598-025-32900-3) contains supplementary material, which is available to authorized users.

## Introduction

Water contamination by microbes and bacteria is one of the most significant challenges in environmental fields, posing a threat to the lives of millions of people worldwide. Predominantly, antibiotics have been used to prevent or treat bacterial infections. However, some of these bacteria become antibiotic-resistant, infecting more humans, and the infections they cause are more difficult to treat compared to those caused by non-resistant bacteria. Hence, antibiotic resistance has emerged as one of the greatest health risks, a global risk to public health, according to the World Health Organization (WHO). The increasing consumption of antimicrobial drugs by humans and animals, as well as the inappropriate prescription of antimicrobial therapy, contribute to the exacerbation of the resistance problem^1^. As a result, new antibacterial agents need to be developed^2,3^. Recently, Covalent organic frameworks (COFs) were used as the drug delivery vehicles because of their biocompatibility and high load capacity, which are connected by non-covalent interactions to guest molecules^4^.

Covalent organic frameworks (COFs) are porous crystalline polymers. They are distinguished by chemical stability, high thermal stability, high crystallinity, and large surface area. Because of their uses in a variety of industries, including gas storage^5–7^, sensors^8^, adsorption^9–11^, mass transport^12^, catalysis^13^, drug delivery^14^, etc., these porous materials have garnered considerable interest. Different strategies have been reported for the synthesis of COF materials, including solvothermal multistep synthesis, microwave synthesis, multicomponent reaction synthesis, mechanochemical synthesis, linker exchange synthesis, etc.^15,16^. The solvents to temperature ratio, crystallization, and reversibility of the reaction, as well as other key factors, can affect the framework of formation^17–19^.

In this study, we report the synthesis of a new COF under green conditions at room temperature. Despite the wide application of COF, they are not widely used for antimicrobial delivery. Therefore, this study observes the antibacterial activity of COFTHB. Herein, we report the green, room-temperature synthesis of a novel azine-linked COF, COFTHB, via the condensation of 1,4-hydrazonmethylbenzene with terephthaldehyde. Unlike previous COFs, COFTHB demonstrates enhanced antibacterial activity against both Gram-negative (*P. aeruginosa*, *E. coli*) and Gram-positive (*E. faecalis*, *S. aureus*) bacteria, outperforming its monomers and standard antibiotics in vitro. COFTHB was characterized using Fourier Transform Infrared spectroscopy (FTIR), Powder X-ray Diffraction (PXRD), Nuclear Magnetic Resonance (NMR), Thermogravimetric analysis (TGA), Brunauer–Emmett–Teller (BET), and Scanning Electron Microscopy (SEM). Molecular docking simulations were conducted to elucidate atomic-level interactions, while Density Functional Theory (DFT) analyses (Frontier Molecular Orbitals (FMO), Electrostatic Potential (ESP), Molecular Electrostatic Potential (MEP) provided insights into its electronic properties and reactivity. This work highlights COFTHB as a promising antibacterial platform for water treatment applications.

## Materials and methods

### Materials

Table 1 provides an overview of the chemical reagents employed in the experiments. It includes details such as the names of the compounds, their empirical or molecular formulas, molecular masses, suppliers, and stated purity levels. Most of the chemicals were sourced from well-known suppliers, predominantly Merck KGaA.

**Table 1:** Empirical formula, CAS registry number, suppliers, and purity of the chemicals.

Component	Empirical formula	Molecular weight (g/mol)	Suppliers	Purity (%)
Terephthaldehyde	[ C_6_H_4_(CHO)_2_]	134.132	Merck KGaA	≥ 95
Hydrazine hydrate	N_2_H_4_	32.0452	Merck KGaA	≥ 95
Benzaldehyde	C_6_H_5_CHO	106.124	Merck KGaA	≥ 99
1,4-dioxane	C_4_H_8_O_2_	88.11	Merck KGaA	≥ 99.5
Ethanol	C_2_H_6_O	46.068	Merck KGaA	≥ 99.5
Acetic acid	C_2_H_4_O_2_	60.052	Merck KGaA	> 99.8

### Synthesis of 1,4-bis(*Z*)-hydrazonnmethyl benzene

**Scheme 1:**
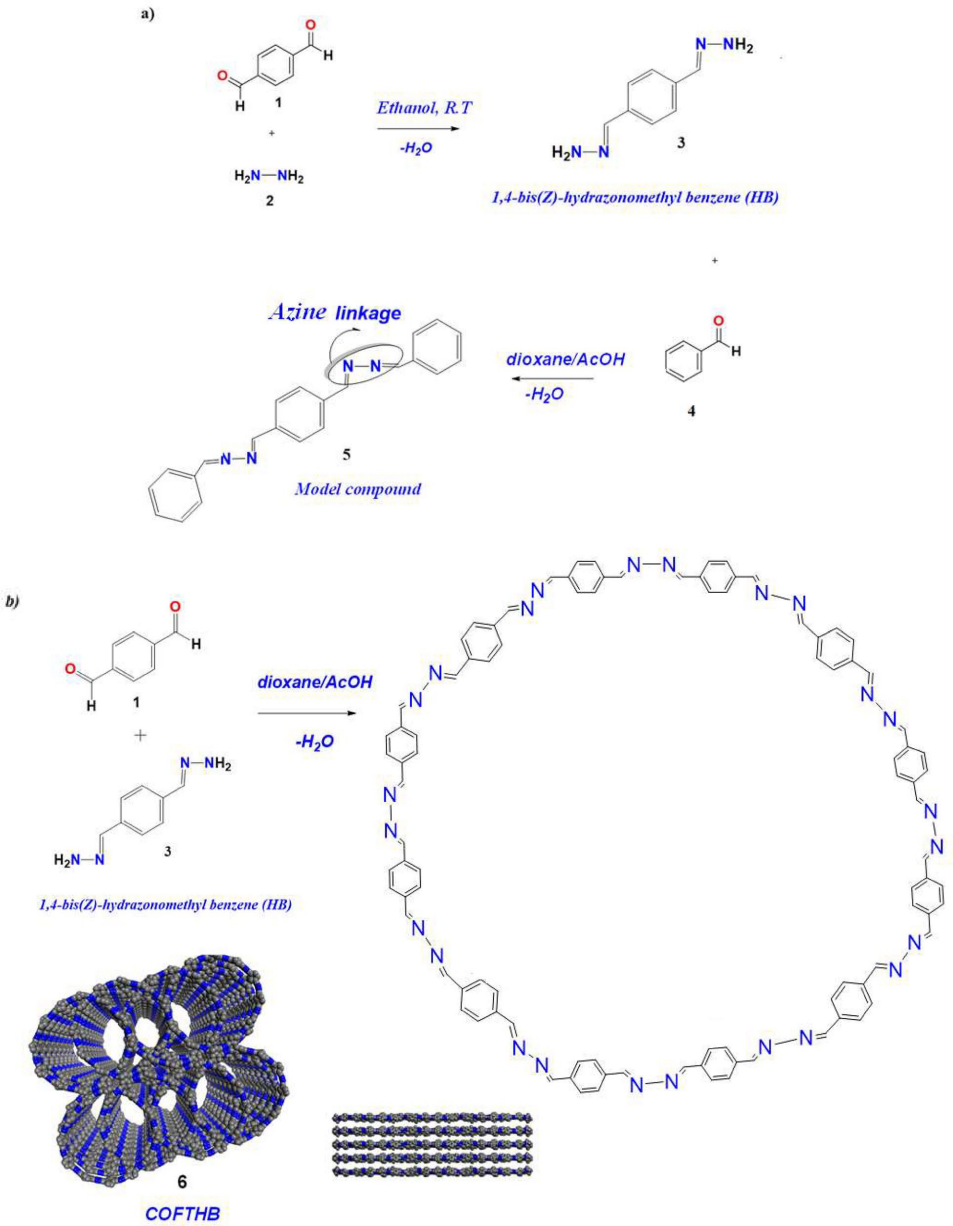
(**a**) Synthesis of 1,4-bis(*Z*)-hydrazonomethyl benzene and model compound, (**b**) Synthesis route of COFTHB and side view of COFTHB.

As shown in Scheme 1a, 1,4-bis (*Z*)-hydrazonnmethyl benzene (HB) was synthesized by condensation of terephthaldehyde and hydrazine hydrate in ethanol at room temperature, and the precipitate formed after 20 min, which increased gradually with time. The reaction was assisted by the polar protic solvent.

### Synthesis of model compound

**Fig. 1:**
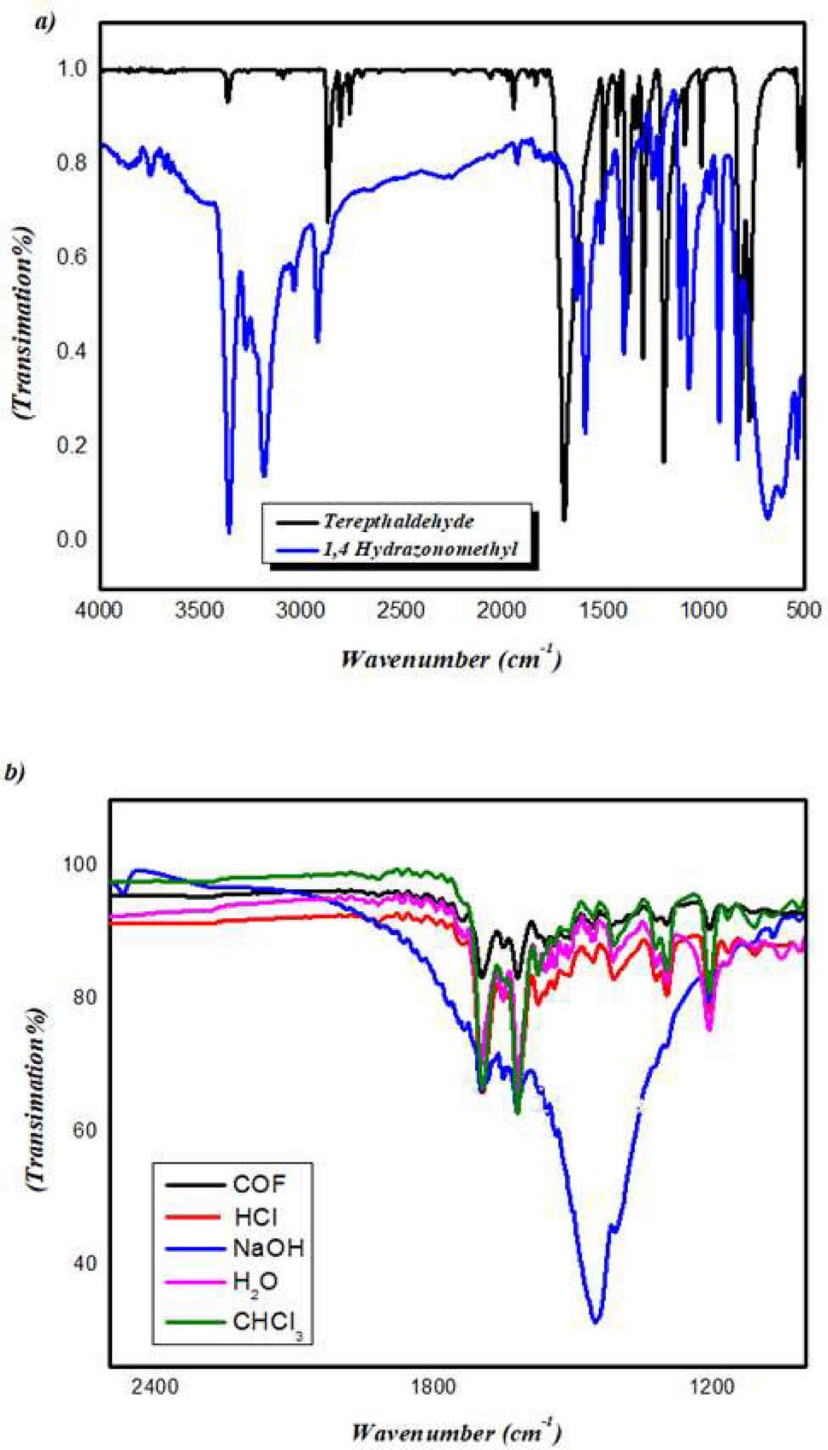
(**a**) FTIR of HB (blue color) and terephthaldehyde (black color); (**b**) FTIR spectrum of the chemical stability of COFTHB under Water, CHCl_3_, NaOH (6M), and HCl (6M).

A model compound (M) was synthesized by the condensation of benzaldehyde and 1,4-bis (*Z*) hydrazonomethyl benzene in the existence of a few drops of catalyst ( 9 M acetic acid) as a in 100 mL of ethanol under reflux then the precipitate was formed (Fig. 1 and S2, in Supporting Information) (Scheme 1a).

### Synthesis of COFTHB

COFTHB was synthesized through the condensation of terephthaldehyde with 1,4-bis(*Z*)-hydrazonomethyl benzene under green conditions in the existence of a few drops of catalyst ( 9 M acetic acid) in 100 ml of dioxane (Scheme 1b) with stirring for 10 h at room temperature, then the yellow precipitate was formed. It was washed with diethyl ether, methanol, and acetone to give COFTHB.

### Characterization

Thermo Fisher Scientific (Waltham, MA) spectrometer was used to measure IR spectra (KBr). ^13^C NMR (*Nuclear magnetic resonance studies)* spectra were measured on a variation GEM 400 MHz spectrometer, where Tetramethyl silane was used as internal standard and the chemical shifts were expressed as δ ppm. The samples were dissolved in deuterated dimethyl sulfoxide (DMSO-*d*_6_). UV/V (*Ultraviolet–Visible spectral measurements*) spectra were performed on a Perkin Elmer Lambda 950 spectrophotometer. TGA (*thermogravimetric analyzer*) was carried out from 100 to 1000 ℃. Scanning electron microscopy (SEM) (*Scanning electron microscopy*) (JOEL, JSM, IT100) was performed to examine the surface morphology. The surface areas and pore volume were determined by N_2_ adsorption/desorption.

### Antibacterial activity

The antibacterial activity of 1,4-bis(*Z*)-hydrazonomethyl benzene, the model compound, tobramycin, and COFTHB were determined by the cut plug method. They were tested against Gram-negative bacteria (*Pseudomonas aeruginosa, Escherichia coli*) and Gram-positive bacteria (*Enterococcus faecalis, Staphylococcus aureus*) in vitro^20^.

#### Minimum inhibitory concentration (MIC)

MIC is the lowest concentration of antibacterial compounds expressed in (mg/L and/or μg/mL), which completely prevents the visible growth of microorganisms under strictly controlled in vitro conditions, after overnight incubation. The antimicrobial properties of compounds were studied in vitro using the dilution tube method (DTM), and the antibiotic tobramycin (TOB) was used as a control (S3, in Supporting Information)^21^. The dilution tube method (DTM) reported by Owuama (2017) was used for the determination of MBC (Minimum bactericidal concentrations) and MIC^22^. This approach has numerous advantages, such as (1) low costs. (2) allows the removal of excess pressure, and (3) can operate for a short time. MBC and MIC for *Escherichia coli, Pseudomonas aeruginosa, Staphylococcus aureus,* and *Enterococcus faecalis* were performed in tubes by diluting twice. The incubation times in the broths containing gentamicin concentrations were tested at 37°C for 18–24 h. The diameters of inhibition zones were measured, and the tube for MIC exhibited growth that became turbid after incubation, while the tube for MBC was still evident.

#### Minimum bactericidal concentrations assay (MBC)

MBC is the lowest concentration of an antibacterial agent required to kill bacteria over a specified and fairly extended period, such as 18 or 24 h, under a specific set of conditions^23^.

### Structural simulation and powder x-ray diffraction analysis

A reflex module implemented in Materials Studio^24^ was employed for determining a crystal structure from powder diffraction data. The structure determination by Accelrys Materials Studio 7.0 software package. The Reflex module determined with the stimulated PXRD patterns P222 space group was chosen for the primitive models in the initial simulations^25^. The crystal class and approximate lattice parameters are derived from the peak positions in the powder diffraction pattern using X-Cell^26,27^.

### Molecular docking analysis

The molecular modeling docking of COFTHB utilizing the Moe program^28^ was used to identify the antimicrobial biological activity to be compatible with experimental analysis. The docking of HB, M, COFTHB, TOB with different protein receptors, such as solution structure of the *E. coli* bacteriophage P1 encoded HOT protein: a homologue of the theta subunit of *E. coli* DNA polymerase III (PDB ID: 1SE7)^29^, the Crystal Structure of P. aeruginosa AmpC (PDB ID: 4WYY)^30^, the *S. aureus* thioredoxin (PDB ID: 2o7k)^31^, and Crystal structure *of E. faecalis* catalase (PDB ID: 1SI8)^32^ to identify their interaction.

### Computational investigation

DFT/WB97XD/6-311(G) basis set was used for theoretical investigation by the Berny method^33^, and using the Gaussian 09W program^34^. The geometry optimization was done without any symmetry requirements. Vibrational Energy Distribution Analysis (VEDA) was used to determine the Potential Energy Distribution (PED), which served as the basis for the wide-ranging vibrational mode assignments^35,36^.

In order to accurately represent the electronic structure and reactivity of COFTHB, a model was developed using a fragment of the overall crystal structure instead of the entire periodic system. The extended COF models could only be optimised using DFT techniques with plane-wave codes (such as VASP, CASTEP, and CP2K) that are beyond the capabilities of Gaussian09W. Therefore, a small segment or oligomer based on the design of multiple azine-linkages (C=N–N=C) was used to represent the COFTHB while representing adequately the local bonded conditions as well as the conjugation pathways and electronic distributions present in the extended COF sheets. This oligomeric representation of the COFTHB maintains the essential electronic properties of azine linkages and the associated aromatic backbone, while allowing for the computational feasibility of conducting molecular-based DFT calculations. To effectively model COFTHB’s electronic structure and reactivity, the authors used a fragment-based representation of the material rather than modelling it as an entire periodic structure. As previously mentioned, fully periodic DFT optimization of extended COFs is only possible with the use of plane-wave codes (VASP, CASTEP, CP2K), which are beyond the scope of Gaussian09W. To create a fragment model for COFTHB that accurately represented its local bonding environment, conjugation pathways, and electronic distributions as an extended 2D sheet, the authors created an oligomer structural model containing a series of azine-linked repeating units connected by the –C=N–N=C– moiety. The oligomer type design retains the essential electronic properties of an azine linkage and aromatic framework while maintaining all properties necessary for molecular DFT analysis. The electronic descriptors from the DFT output EHOMO (Energy of the Highest Occupied Molecular Orbital energy), ELUMO (Energy of the Lowest Unoccupied Molecular Orbital), ΔEg, electronegativity (χ), chemical hardness (η), softness (σ), electrophility index (ω), and maximum electron transfer (ΔNmax) were calculated using the output from DFT. The molecular electrostatic potential and electron density maps visualise the local electron-rich and electron-poor regions relevant to bacterial interactions. Additional support for the assignment of vibrational modes and energy decomposition was provided by VEDA (Vibrational Energy Distribution Analysis) analysis. The COFTHB model is an oligomeric repeating unit, and therefore, the results provide insight into the local electronic properties of the azine-linked framework, but these results do not reflect a full periodic DFT calculation.

## Results and discussion

### Chemistry

#### Synthesis of 1,4-bis(Z)-hydrazonnmethyl benzene (HB)

For the successful preparation of 1,4-bis(*Z*)-hydrazonomethyl benzene (HB), the reaction condition was optimized. The molar ratio of monomer, temperature, and organic solvent plays an important role in this method. The molar ratio of the monomers should be (1:2 mol) of aldehyde (terephthaldehyde) to amine (hydrazine hydrate). Only the polar protic solvent can be used to assist the formation of the free amine as (=N-NH_2_). Also, the temperature is necessary to get free amine (NH_2_).

#### Synthesis of model compound (M)

A model compound (M) was synthesized by the molar ratio of monomer (benzaldehyde: 1,4-bis (*Z*) hydrazonomethyl benzene) (1:1) in the presence of acetic acid as a catalyst in ethanol under reflux for 3h to afford a yellow precipitate.

#### Synthesis of COFTHB

COFTHB was synthesized condensation of terephthaldehyde and 1,4-hydrazonmethyl benzene with stirring under green conditions (Scheme 1) (Schiff Base reaction). The monomers were dissolved in dioxane, and then the reaction was going to stir for several hours, and a precipitate was formed by the formation of strong azine linkages between the units.

### FT-IR spectral analysis

#### FT-IR spectral analysis of 1,4-bis(Z)-hydrazonomethyl benzene

For FT-IR spectroscopy, it was confirmed by the absence of carbonyl C=O (1689 cm^−1^) of terephthaldehyde, the presence of amine group of peaks of HB at (3349, 3187 cm^−1^), and the formation of imine functional groups C=N at (1607 cm^−1^) (Fig. 1a).

#### FT-IR spectral analysis of model compound

The model compound exhibited the absence of carbonyl C=O (1695 cm^−1^) of benzaldehyde, amine group of peaks of HB at (3349, 3187 cm^−1^), and formation of the –C=N band at 1613 cm^−1^ Fig. 1a.

#### FT-IR spectral analysis of COFTHB

For the first glimpse, the reaction of HB and terephthaldehyde was expected to form a triazine organic framework. However, upon close inspection, we found the occurrence of HB containing C=N–NH_2_, which qualifies it to generate an azine-linked conjugate structure at room temperature. The FT-IR spectrum reveals the absence of carbonyl group C=O at (1711 cm^−1^), which represents the terephthaldehyde group and the amine group (NH_2_) at (3349, 3187 cm^−1^) occurring on the 1,4-bis(*Z*)-hydrazonomethyl benzene. Moreover, the formation of imine functional groups C=N at (1694 cm^−1^), and phenyl group at (1612 cm^−1^) were confirmed as revealed in Fig. 1b. The C = N stretching band appears at ~ 1694 cm⁻^1^, which is higher than typical imine values (1600–1650 cm⁻^1^). This blue shift is attributed to the strong electron-withdrawing environment of the azine linkage, extended conjugation within the framework, and possible interlayer hydrogen-bond interactions, all of which increase the effective C=N bond order. Similar shifts have been reported for other azine-linked COFs. 2D COFTHB showed high stability in water, HCl (6M), and CHCl_3_ were stable for one week at room temperature. In contrast, the dissociation of the azine linkage was exhibited in the presence of NaOH (6M) and showed a new sharp absorption band at 1453 cm^−1^_,_ comparable with the FT-IR of COFTHB. It was insoluble in water as well as other common organic solvents, such as ethanol, hexane, acetone, CHCl_3_, *N, N*-dimethylformamide, and tetrahydrofuran. Figure 1b.

### ^1^H and ^13^C Nuclear magnetic resonance

#### Nuclear magnetic resonance of 1,4-bis(Z)-hydrazonomethyl benzene

**Fig. 2:**
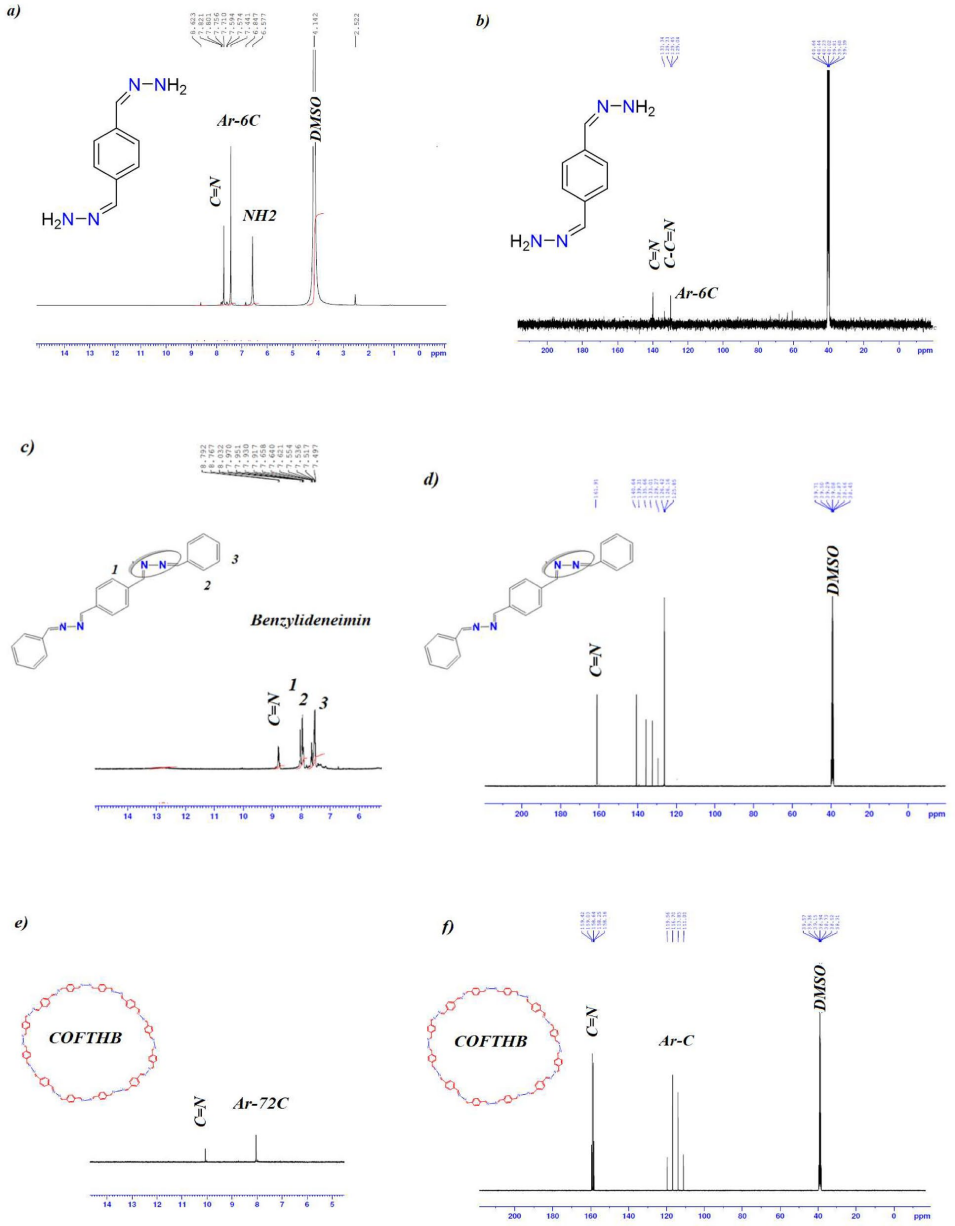
(**a**) ^11^H NMR of HB; (**b**) ^13^C NMR of HB; (**c**) ^1^H NMR of M; (**d**) ^13^C NMR of M; (**e**) ^1^H NMR of COFTHB; and^13^C NMR of COFTHB.

^1^H NMR spectrum of HB showed a specific signal for C=N at 7.80 ppm and a broad signal of NH_2_ at 6.57 ppm (Fig. 2a). For ^13^C NMR , the C=N signal was displayed at 140.64 ppm (Fig. 2b).

#### Nuclear magnetic resonance of model compound

^1^H NMR of model compound exhibited a signal (CH=N) at 8.79 while the two-terminal aromatic benzene at 7.49 (8H, m, Ar–H) and the internal aromatic benzene at 8.03 (4H, s, Ar–H (Fig. 2c). For ^13^C NMR, the C=N signal was displayed at 161.92 ppm (Fig. 2d).

#### Nuclear magnetic resonance of COFTHB

^1^H NMR spectrum of COFTHB exhibited an imine group (–C=N) at 10.11ppm (Fig. 2e); ^13^C NMR spectrum showed a band for the azine bond (C=N) at 158.73 ppm. Fig. 2f reveals the absence of (C=O) of terephthaldehyde ~ 191.57 ppm. Other additional signals refer to the symmetrical aromatic carbons of benzene. Elemental analysis of the COFTHB showed the presence of C, H, and N at 73.18, 4.18, and 21.42%, respectively, which were close to the calculated values of C (73.83 %), H (4.65 %), and N (21.52 %) expected for this kind of reaction.

### UV-V spectrum

**Fig. 3:**
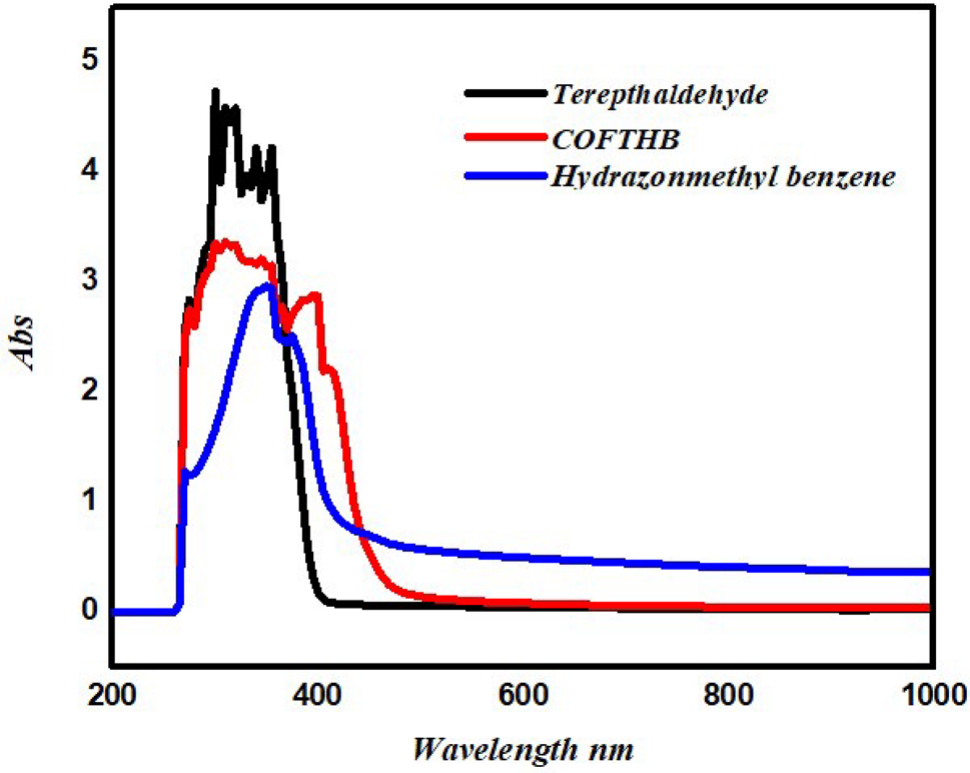
(**a**) UV-V spectrum of terephthaldehyde, HB, and COFTHB.

UV-V spectrum of terephthaldehyde was 298 nm, while HB was 349 and 377 nm (Fig. 3) according to π–π and n–π transition. UV–V spectrum of COFTHB shifted to higher wavelength 317, and 404 nm based on the π–π and n–π transition, due to a relatively larger conjugated COF system (Fig. 3).

### Powder X-ray diffraction of COFTHB

**Fig. 4:**
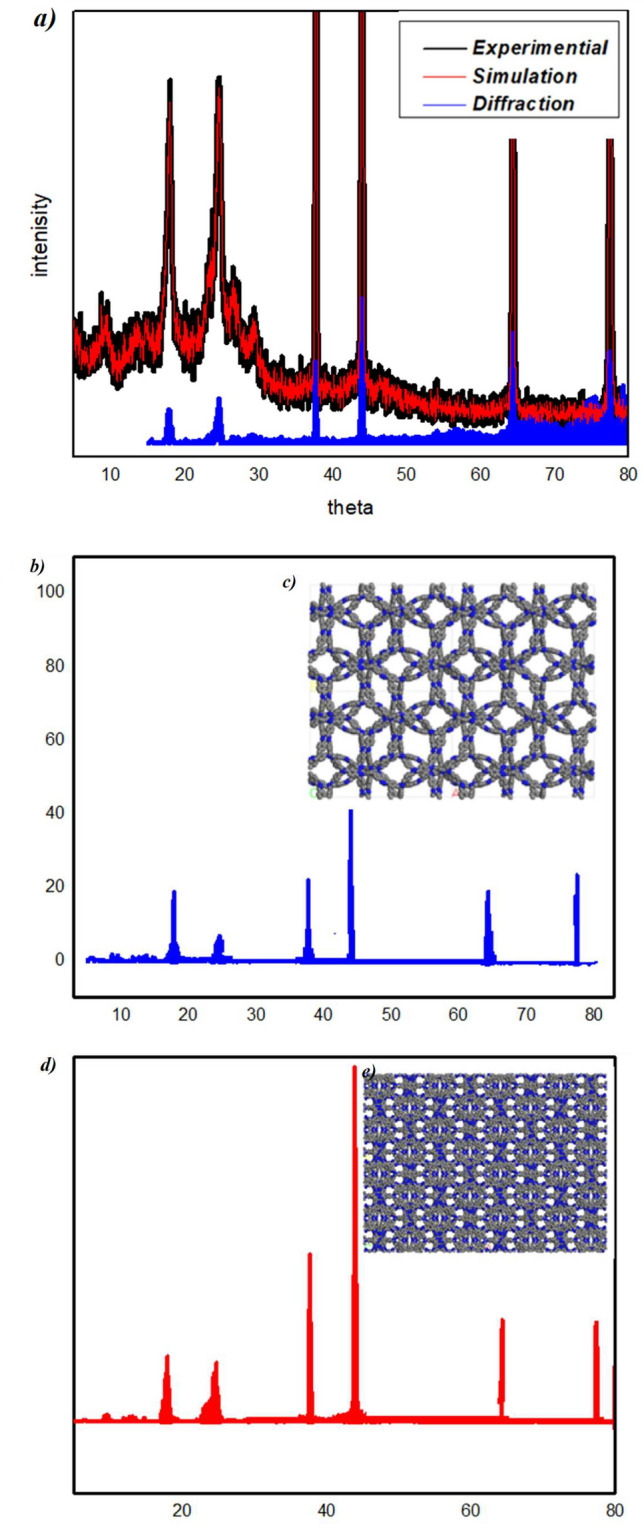
(**a**) PXRD patterns of the COFTHB with the experimental pattern in black, the Pawley refinement in red, and their difference in blue; (**b**) Simulated PXRD pattern for AA structure; (**c**) AA unit-cell structure; (**d**) Simulated PXRD pattern for AB structure; (**e**) AB unit-cell structure.

From a topological viewpoint, a combination of symmetric C2 monomers was supposed to generate two crystallographic systems: one hexagonal system with two different kinds of pores and the other is an orthorhombic system that has one kind of pore (Fig. 4a). Furthermore, there are two types of orthorhombic systems: staggered and eclipsed^37^. 1,4-bis(*Z*)-hydrazonomethyl benzene (HB) and terephthaldehyde were selected as the C2 symmetric monomer.

The crystalline structure of the COFTHB was examined by PXRD measurements. The COFTHB showed a strong peak at 37.64, 43.89, 64.26, and 77.36. The low intensity of the first peak at 2ɵ =  ~ 9.5° revealed the porosity of COF and low crystallinity. The slight broad peak at higher 2ɵ (~ 17.55 and 24.75) is mainly due to π–π stacking between the COF layers, which corresponds to the (001) plane (Fig. 4a). The d spacing of COFTHB was found to be 3.95 Å, and it was smaller than previously reported 2D-COF layers. The Pawley refined pattern was used to approve the experimental diffraction peaks (Fig. 4). Using Materials Studio, version 7.0, the unit cell parameters were computed based on 2D sheets following geometrical energy minimization. The theoretical investigation was performed using the P222 space group with a = b = 37.0 Å, c = 8.7 Å, and a = b = $\gamma$  = 90° of COF found to be very close to the experimental structure with Rp = 5.59% and Rwp = 8.18%. The PXRD pattern of an eclipsed stacking AA and the AB was found to be quite similar to the experimentally observed profile. The simulation of the PXRD pattern of the unit-cell structures was observed in an eclipsed stacking AA and the AB staggered, which were close to the experimentally observed profile (Fig. 4b, c) and (Fig. 4d, e).

### Scanning electron microscope (SEM)

**Fig. 5:**
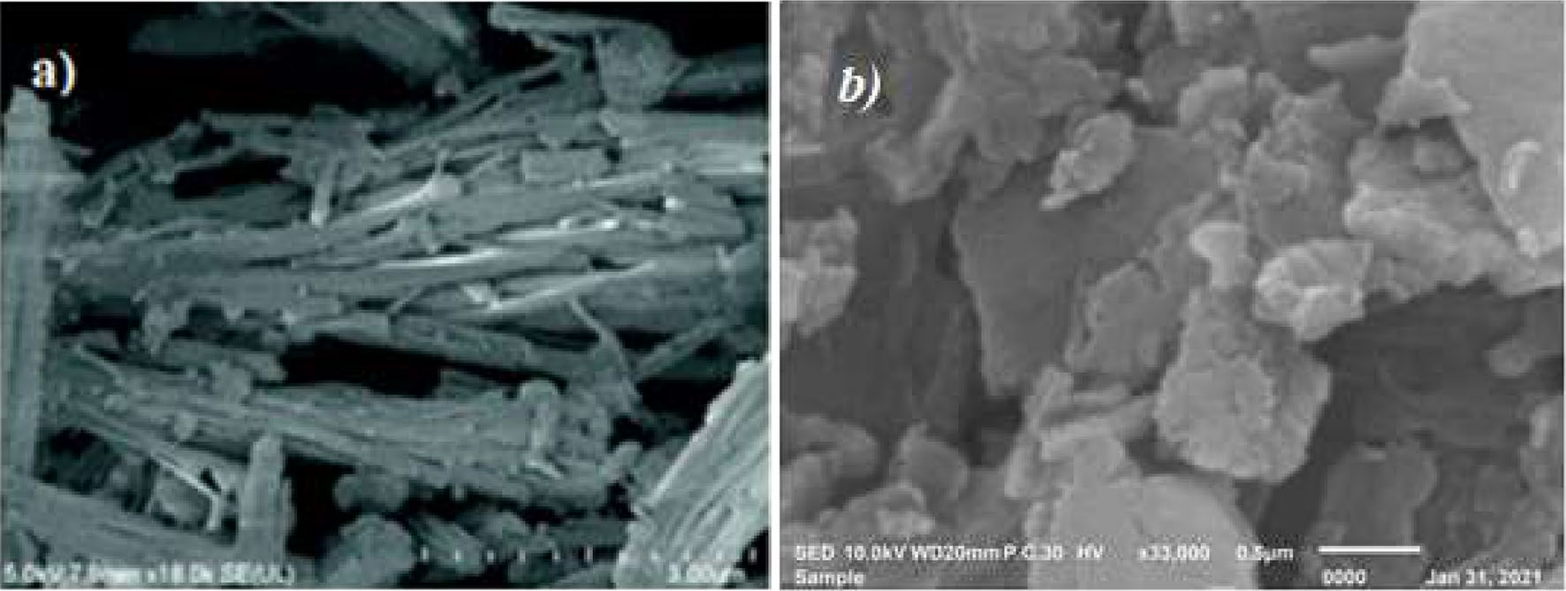
(**a**) SEM of Terephthaldehyde; (**b**) SEM of COFTHB 0.5 μm.

Scanning electron microscope (SEM) with a high magnification of 140kX. SEM images reveal only one morphological structure of COFTHB (Fig. 5). The surface morphology of COFTHB confirms the uniformity in the porosity. The terephthaldehyde morphology, which appeared as rods with long needles, was quite different from that of COFTHB (Fig. 5a)^38^. This difference in morphology proves the possibility of copolymerizing terephthaldehyde with 1,4-bis(*Z*)-hydrazonomethyl benzene to obtain COFTHB (Fig. 5b).

### Thermal Stability for COFTHB (TGA)

Thermogravimetric analysis (TGA) of COF-THB was performed under a nitrogen atmosphere and revealed excellent thermal stability up to 629 °C. As the temperature increased from 0 to 181.5 °C, a weight loss of approximately 6.9% was observed, which was attributed to the desorption of adsorbed water. A more significant weight loss of about 37.9% occurred between 401 and 629 °C, corresponding to the breakdown of the COF framework (Fig. S5). At higher temperatures, the aromatic structure underwent carbonization, leading to further damage and decomposition of the molecular network. Overall, COF-THB demonstrated superior thermal stability up to 629 °C, surpassing that of other azine-linked COFs such as HEX-COF-1(250 °C)^39^, Py-Azine COF (250 °C)^40^, and ACOF (300 °C)^5^.

### Adsorption isotherm

**Fig. 6:**
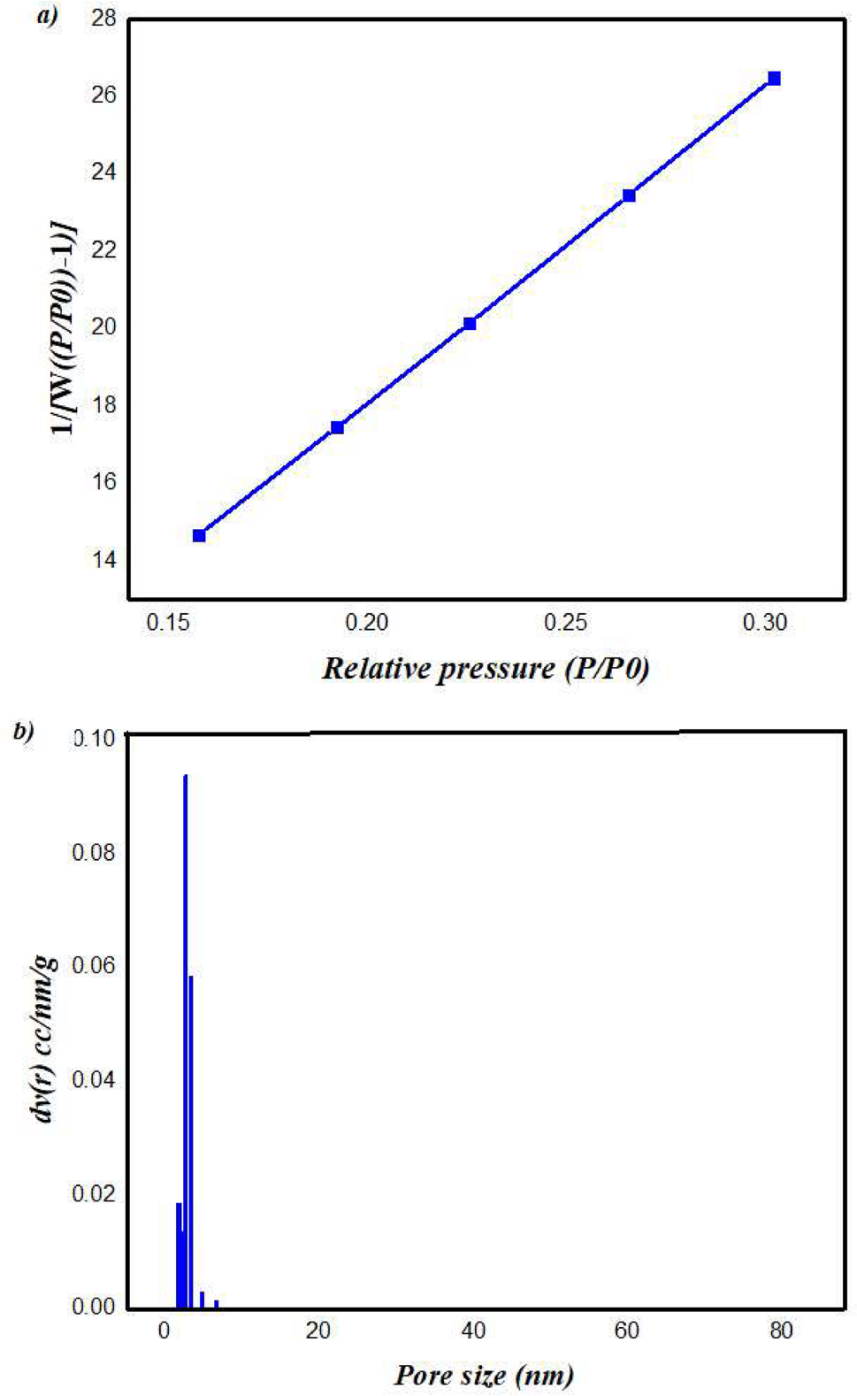
(**a**) Surface area (BET) of COFTHB; (**b**) Pore size distribution of COFTHB.

According to the International Union of Pure and Applied Chemistry^41^, the sorption curve of the COFTHB displayed in Fig. S6 demonstrates IV-N_2_ sorption isotherm features in the P/P0 = 0 to 0.1 range. At relatively high pressure, hysteresis can be seen in the COFTHB isotherm, which is linked to the irreversible uptake of nitrogen in the pores. Moreover, COFTHB exhibits a surface area of 68.57m^2^ g^−1^ (Fig. 6a). The theoretical surface area was estimated to be 1546.08 m^2^ /g. Azine-linked COFs are known to suffer from pore collapse, strong interlayer interactions, and limited reversibility during polymerization, often resulting in surface areas that are far below the theoretical maximum. Several reported azine COFs show similarly low BET areas. The COFTHB surface area was greater than the previously published COF values for BT-DG_Cl_ (3 m^2^/g), HBF (30 m^2^/g), TpBD1 (35 m^2^/g), TpPa-2 1 (56 m^2^/g), and TpPa-1 (61 m^2^/g)^42–45^. This trend is intrinsic to the azine (–C=N–N=) linkage, which promotes layer distortion, π–π stacking, and partial pore blocking, particularly in non-solvothermal syntheses. The pore size of COFTHB was 3.68 nm, which demonstrated a mesoporous nature of the network (Fig. 6b) with a total pore volume of 0.113 cm^3^ g^−1^. The value obtained for COFTHB aligns well with the structural characteristics of azine-linked frameworks. Furthermore, the room-temperature green synthesis employed in this work intentionally avoids harsh solvothermal conditions, which typically enhance crystallinity but are not environmentally friendly; consequently, some reduction in porosity is expected. Importantly, despite its modest BET surface area, COFTHB maintains excellent chemical/thermal stability and superior antibacterial performance, indicating that functional activity arises mainly from surface chemistry and azine-based electrostatic interactions rather than from high porosity alone.

### Biological study

#### Antibacterial activity

**Fig. 7:**
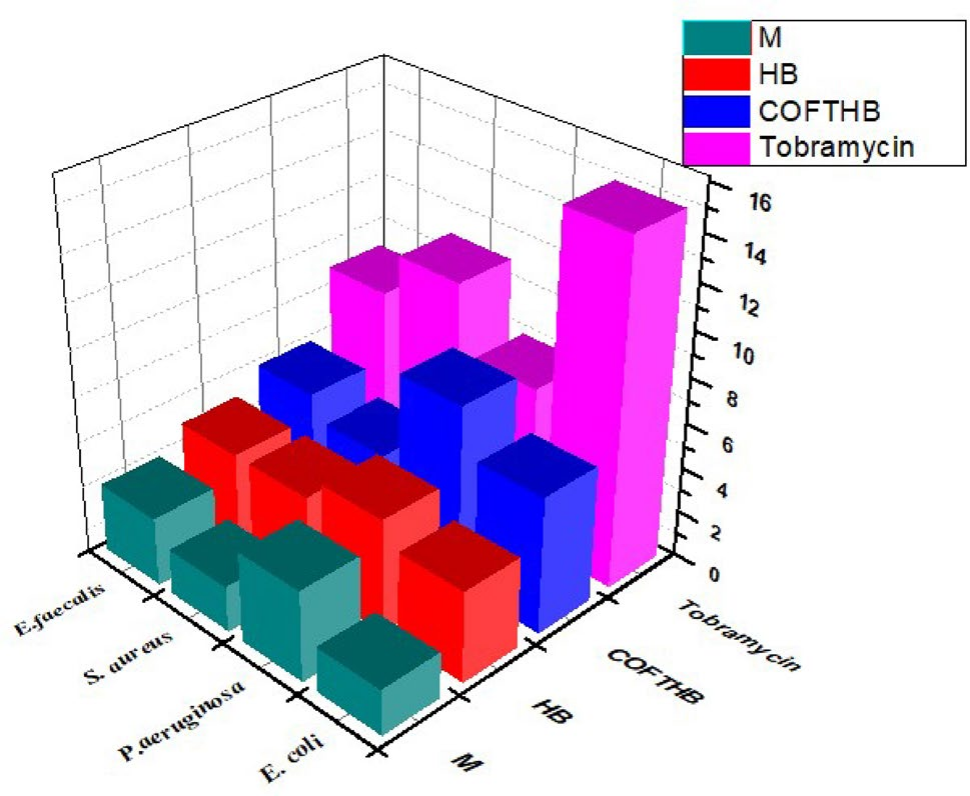
The inhibition zone diameter values of 1,4-bis(*Z*)-hydrazonomethyl benzene (HB), model compound (M), and COFTBH against Gram-positive and Gram-negative (mm).

In this study, 1,4-bis(*Z*)-hydrazonomethyl benzene (HB), model compound (M), tobramycin (TOB), and COFTHB were tested against Gram-positive bacteria (*S. aureus, E. faecalis*) and Gram − negative bacteria (*E. coli, P. aeuroguinsa*). The inhibition zone diameter of HB, M, TOB, and COFTBH for *E. coli, P. aeuroguinsa, S. aureus,* and *E. faecalis* were (2.0, 4.0, 15.0, and 6.0), (4.0, 5.0, 7.0, and 8.0), (2.0, 4.0, 10.0, and 4.0), and (3.0, 4.0, 8.0, and 5.0) mm, respectively (Fig. 7).

TOB is an aminoglycoside antibiotic material with a broad spectrum of in vitro antibacterial activity (Fig. 7). The inhibition growth of microorganisms of COFTBH may be through electrostatic interaction, hydrogen bond, Vander Waals, and hydrophobic bond. These interactions can destroy the membrane of bacteria and disrupt their cellular components. Hence, it causes the bacteria to die^46^.

**Fig. 8:**
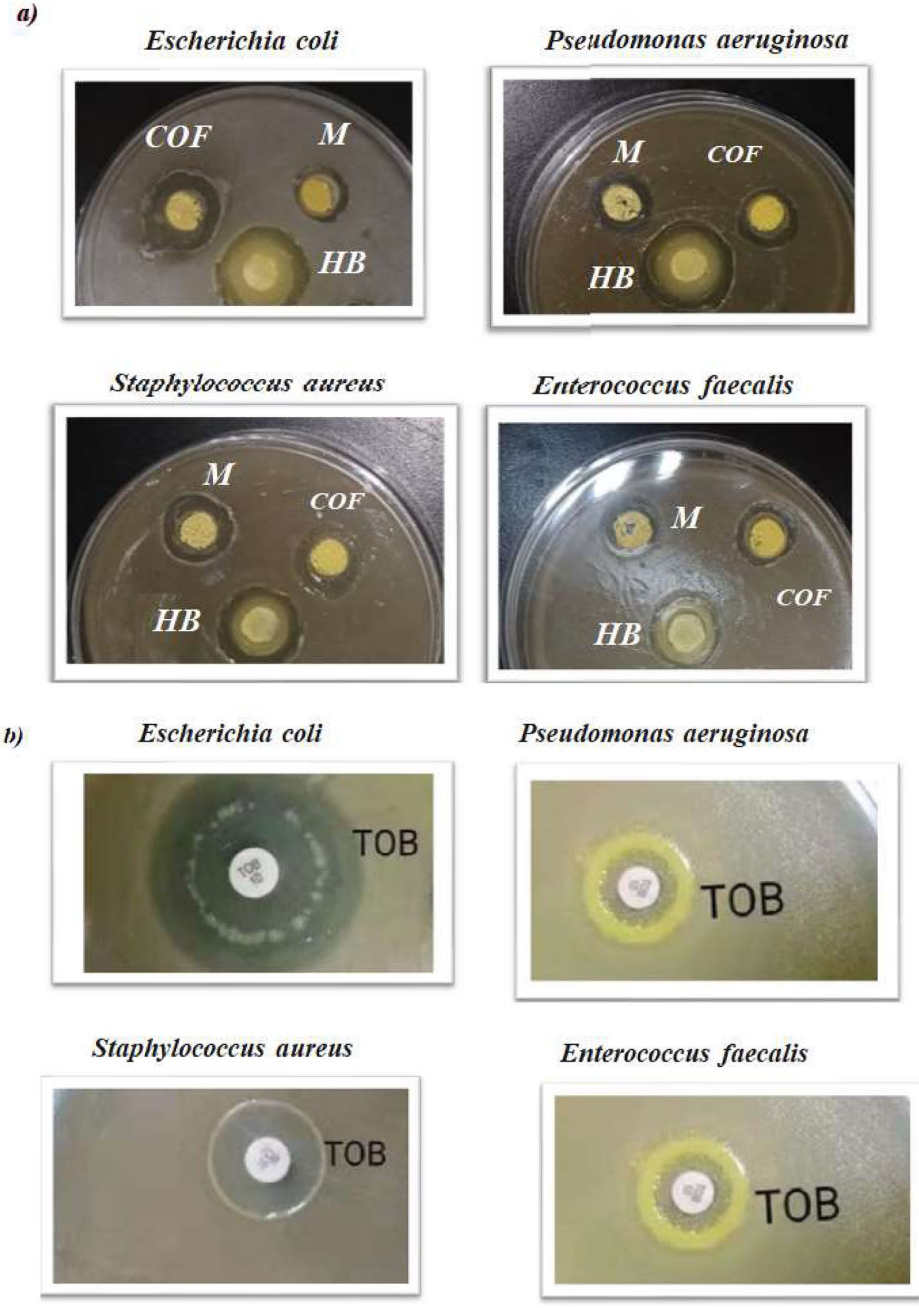
The diameter of the inhibition zone images of 1,4-bis(*Z*)-hydrazonomethyl benzene (HB), model compound (M), COFTHB, and Tobramycin (TOB) against E. Coli P. Aeruginosa S. aureus, and E. Faecalis bacteria in vitro.

COFTBH exhibited a higher antibacterial activity than 1,4-bis(*Z*)-hydrazonomethyl benzene (HB), model compound (M) (Fig. 8) due to (1) larger conjugation; (2) abundant of azine functional group; (3) electrostatic interactions between positively charged of imine group (C^+δ^–N^+δ^) units and phospholipid bilayers, this play a key role for the antibacterial response of COF; 4) hydrogen bonding interactions between the azine groups of COFTHB and phospholipids might have contributed to the antibacterial mechanism as well^47^.

The inhibition of the growth of microorganisms by COFTHB, as displayed in Fig. 8, was probably through: (1) cell membrane depolarization, the azine linkage (C=N) in the COFTHB can polarize the carbon atoms that bear the positive charge, while N bears the negative charge. This forms a coordination bond between the lone pair of (N–N) in the azine linkage and causes an electrostatic attraction of the negatively charged nitrogen. (2) This can inhibit protein synthesis, depending on the adsorption concentration, which can result in the coagulation and precipitation of cytoplasmic proteins; (3) or inhibit cell wall synthesis through electrostatic interactions, hydrogen bridges, probably by a coordination bond or hydrophobic bond. Other factors that might affect the inhibition efficiency are the concentration and the surface area of the COFTHB.

#### Minimum Bactericidal Concentration (MBC)and Minimum inhibitory concentration (MIC)

**Fig. 9:**
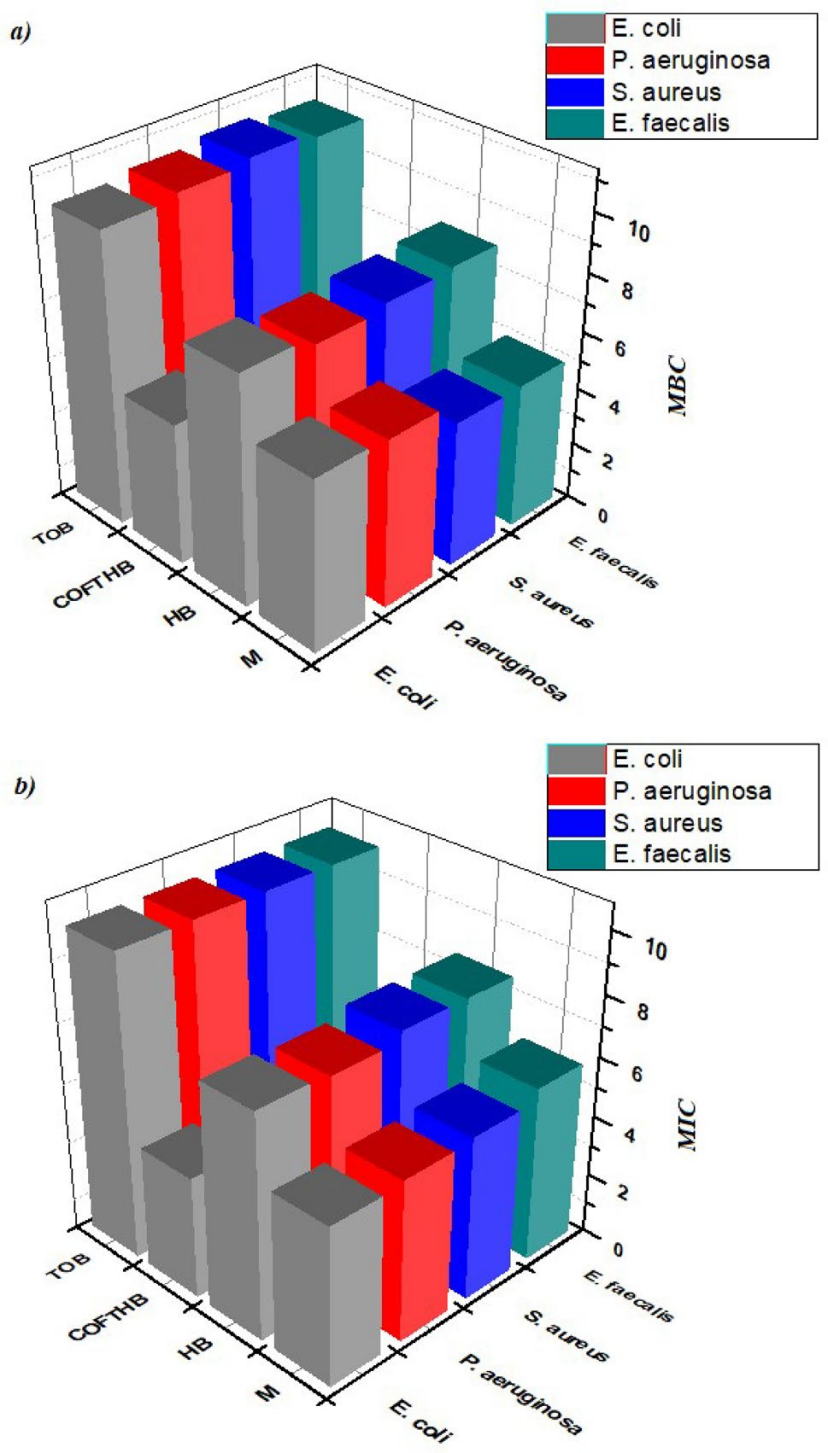
(**a**) MIC and (**b**) MBC values for E. Coli P. Aeruginosa S. aureus, and E. Faecalis bacteria (mg/ml).

COFTHB exhibited higher sensitivity than TOB (Table 2). Only ~ 4 mg/ml of COFTHB was used to inhibit the growth of bacteria. In comparison, ~ 7, 5, 10 mg/ml of HB, M, and TOB were used, respectively (Fig. 9). While COFTHB showed lower MIC values than Tobramycin, its inhibition zones were smaller due to limited diffusion of the solid COF material in agar, whereas TOB diffuses more readily because of its small molecular size and high solubility. Therefore, the “higher sensitivity” conclusion refers specifically to MIC/MBC performance, not to agar-diffusion results. COFTHB showed a higher sensitivity for gram-positive than gram-positive, this may be referred to as (1) the coordination bond or electrostatic interactions between the lone pair of nitrogen in azine (C=N–N) of COF and highly positive charge of gram-positive bacteria on the cell surface which can play an important role for the antibacterial response; (2) the hydrogen bonding interactions between the azine groups of COF and the phospholipids which could as well contribute to the antibacterial mechanism; (3) higher conjugation lead to the higher number of negative charge that represents in azine bond; (4) Also, the surface area of COF may contribute to higher sensitivity.

**Table 2:** Inhibition zone diameters (IZ), Minimum Inhibitory Concentration (MIC), and Minimum Bactericidal Concentration (MBC) of HB, M, COFTHB, and TOB against tested antibacterial strains.

	HB			*M*			*COFTHB*			*Tobramycin antibiotic*		
	*IZ (mm)*	*MIC (mg/ml)*	*MBC (mg/ml)*	*IZ (mg/ml)*	*MIC (mg/ml)*	*MBC (mg/ml)*	*IZ (mm))*	*MIC (mg/ml)*	*MBC (mg/ml)*	*IZ (mg/ml)*	*MIC (mg/ml)*	*MBC (mg/ml)*
*E. coli*	4	**7.4**	7.9	2	**5.2**	5.9	6	**4**	4.9	15.00	10.0	10.1
*P. aeruginosa*	5	**7.3**	7.6	4	**5.3**	5.8	8	**4.2**	4.9	7.00	10.0	10.3
*S. aureus*	4	**7.6**	7.8	2	**5.4**	5	4	**4.5**	4	10.00	10.0	10.5
*E. Faecalis*	4	**7.6**	7.9	3	**5.8**	5	5	**4.6**	3.9	8.00	10.0	10.3

**Fig. 10:**
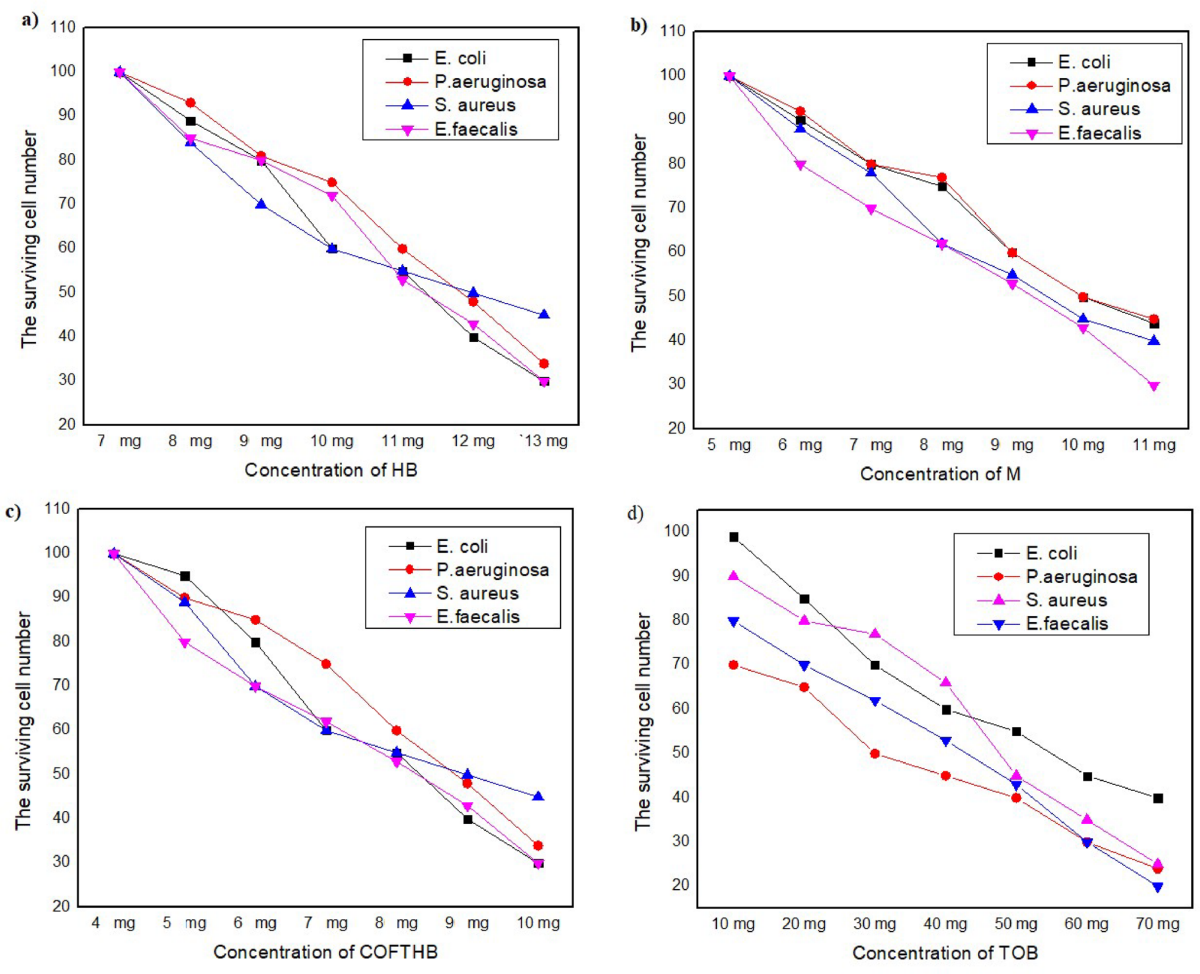
(**a**–**d**) The cell-survival curve of HB, M, TOB, and COFTHB versus concentration against gram-positive and gram-negative bacteria.

To study the effect of HB, M, TOB, and COFTHB concentration quantitatively on the survival of microorganisms, different concentrations were used. It is important to note that for all the material considered in this study, the surviving cell number decreases as the concentration decreases (Fig. 10). It is evident that by increasing the concentration, the inhibition of bacteria increases. At 10 mg/ml (Fig. 10b), COFTHB showed inhibition of bacteria reached 70% (Fig. 10c) while TOB exhibited inhibition of bacteria by 80% (Fig. 10d). In the case of HB and M, at 7 and 5 mg/ml, they inhibited the growth of bacteria by 100%, then decreased up to 10% respectively (Fig. 10a and b). Thus, demonstrating that a COFTHB had a much better performance at lower concentration compared to other materials.

#### Antibacterial mechanism

Although COFTHB has demonstrated high levels of antibacterial activity through MIC, MBC, and inhibition-zone studies, as well as by computational docking and DFT analysis, we acknowledge that our existing knowledge regarding COFTHB’s mechanisms of action is still being developed. The proposed mechanisms of action, through the electrostatic attraction between azine linkages and bacterial membranes, hydrogen bonding, membrane depolarization, and potentially disrupting cellular components, are all based on chemical structure–activity relationships and respective computational docking phenomena. However, no direct experimental assessments have yet been performed to substantiate this proposed mechanism. It is therefore advisable to interpret the current data as an experimental model of COFTHB action, while additional studies will be needed to clarify how COFTHB primarily acts (via membrane disruption alone, intracellular components, or multiple avenues of bactericidal activity).

### Molecular docking studies

**Fig. 11:**
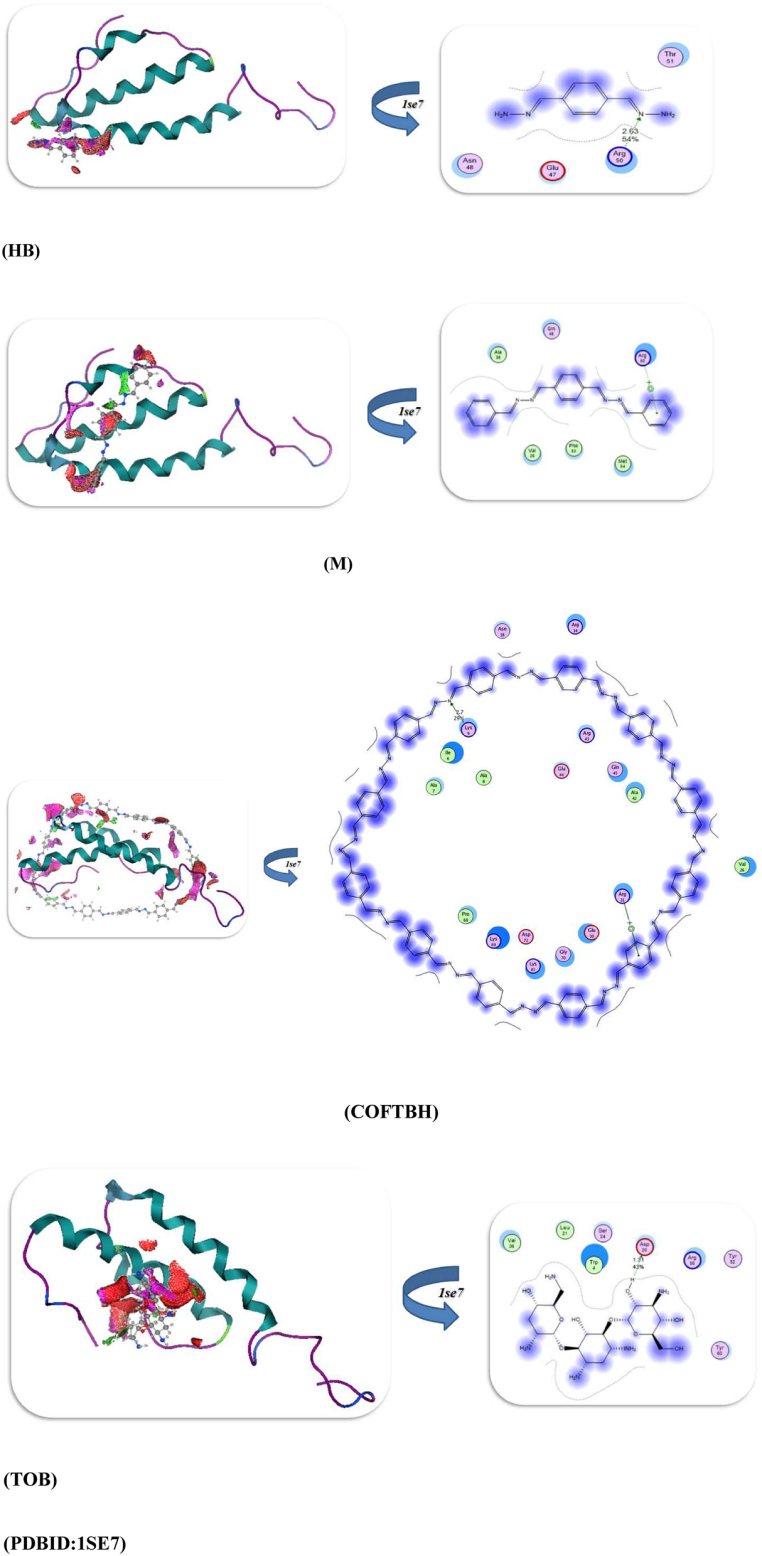
The docking interaction of HB, M, COFTBH, and TOB; respectively with PDBID:1SE7.

The performance of the docking simulation using the MOE program^28^ was used to confirm the biological action with the attachment of different protein sites to its amino acids. So in this study, we docked the HB, M, COFTHB, and TOB with different proteins, such as the Solution structure of the *E. coli* bacteriophage P1 encoded HOT protein: a homologue of the theta subunit of *E. coli* DNA polymerase III (PDB ID: 1SE7)^29^. The Crystal Structure of *P. aeruginosa* AmpC (PDB ID: 4WYY)^30^, the S. aureus thioredoxin (PDB ID: 2o7k)^31^, and Crystal structure of E. faecalis catalase (PDB ID: 1SI8)^32^, as displayed in Table 3. Firstly, the docking results of HB, M, COFTHB, and TOB with (PDB ID: 1SE7) showed binding energy − 8.7321, − 7.5543, − 7.7397, and − 10.6684Kcal/mol; respectively, and with the lowest bond length with TOB (1.31Å) and make more hydrogen bonding interaction with proteins Tyr 52, Arg 56, Asp 20, Ser 24, Trp 4, Leu 21, Val 26 rather than other compounds but also the HB showed hydrogen bonding with Thr 51, Arg 50, Glu 47, Asn 48 and shortage distance 2.63Å, while the M and COFTBH were showed electrostatic interactions between amino acids Arg 50, Gln 48, Ala 38, Val 25, MeI 54 and Ala 42, Arg 31, Gln 45, Arg 43, Glu 44, Ala 8, Ala7, Lys 69, Asp 72 from C=N linkage as displayed in Table 3 and Fig. 11.

**Table 3:** The molecular docking approaches of COFTHB with different proteins.

*E. coli (PDB:1SE7)*					*S. pneumoniae (PDB:4WYY)*		
	Energy affinity (kcal/mol)	Distance(Å)	Amino acids		Energy affinity (kcal/mol)	Distance(Å)	Amino acids
*HB*	− 8.7321	2.63Å	*Thr 51, Arg 50, Glu 47, Asn 48*	*HB*	− 9.5418	2.52Å	Gly 129, Ser 131, Arg 114, Asp 134, Leu 133, Arg 327
*M*	− 7.5543	3.2Å	*Arg 50, Gln 48, Ala 38, Val 25, MeI 54*	*M*	− 7.7327	2.9Å	Tyr 283, Leu 111, Leu 104, Leu 113, Asp 134, Asp 112, Ser 131
*COFTHB*	− 7.7397	3.5Å	*Ala 42, Arg 31, Gln 45, Arg 43, Glu 44, Ala 8, Ala7, Lys 69, Asp 72*	*COFTHB*	− 8.7167	3.5Å	Leu 133, Leu 111, Asp 112, Arg 114, Asp 187, Ser 111
*TOB*	− 10.6684	1.31Å	*Tyr 52, Arg 56, Asp 20, Ser 24, Trp 4, Leu 21, Val 26*	*TOB*	− 10.7352	1.86, 2.82Å	Val 239, Pro 241, Ala 246, Gly 240, Asp 245, Lys 183

*S. aureus (PDB:2O7K)*				*E. faecalis (PDB:1si8)*			
	Energy affinity (kcal/mol)	Distance(Å)	Amino acids		Energy affinity (kcal/mol)	Distance(Å)	Amino acids
*HB*	− 10.8992	1.64,2.81Å	Ile72, Trp28, Ser 71, Met 70	*HB*	− 8.7027	1.53Å	Asp 457, Arg 454, Leu 458, Pro 169
*M*	− 7.0767	3.8Å	Ile72, Ser 71, Phe 90, Pro 31, Gly 89	*M*	− 7.0554	3.2Å	Ser 411, Arg 461, Gln 414, His 416, Pro 165, Asp 136,
*COFTHB*	− 8.1491	4.2Å	His 103, Asp 85, Val84, Lys86, Trp 28, Asp 58, Glu 59Thr 7, Ala9, Asp10	*COFTHB*	− 7.8712	2.72Å	Gln4, Leu 6, His 5, Gln 36, Phe 372, Leu 352, Met 170, Val 354
*TOB*	− 11.3676	1.59,2.54Å	Tyr67, Gln 82, Lys66, Asp 12, Asp80, Glu 16	*TOB*	− 10.4667	1.57,1.72,2.44Å	His 167, Glu 170, Pro 169, Lys 139, His 416, Ser 168, Gln 414, Arg 461

**Fig. 12:**
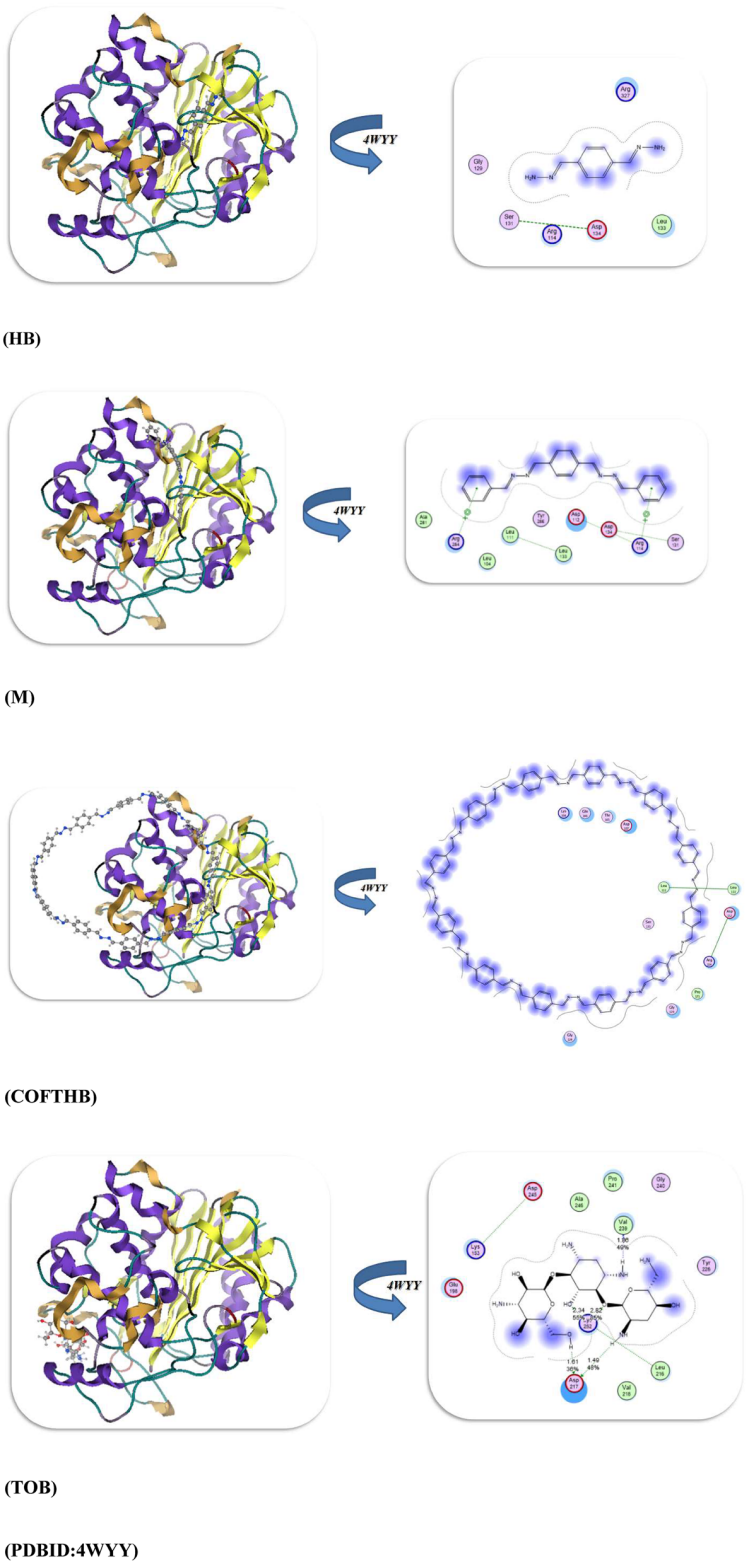
Docking interaction of compounds with PDBID:4WYY.

Furthermore, the docking interaction of HB, M, COFTBH, and TOB with PDBID:4WYY showed also the stability of TOB and HB rather than other compounds with − 10.7352 kcal/mol, − 9.5418 kcal/mol and binds with proteins through hydrogen bonding Val 239, Pro 241, Ala 246, Gly 240, Asp 245, Lys 183 and Gly 129, Ser 131, Arg 114, Asp 134, Leu 133, Arg 327 with smallest length range 1.86–2.82Å, then the COFTBH and M showed electrostatic interaction with amino acids Leu 133, Leu 111, Asp 112, Arg 114, Asp 187, Ser 111, Tyr 283, Leu 111, Leu 104, Leu 113, Asp 134, Asp 112, Ser 131 and with the lowest energy − 8.7167 kcal/mol, − 7.7327; respectively which is confirmed of experimental results as showed in Fig. 12.

**Fig. 13:**
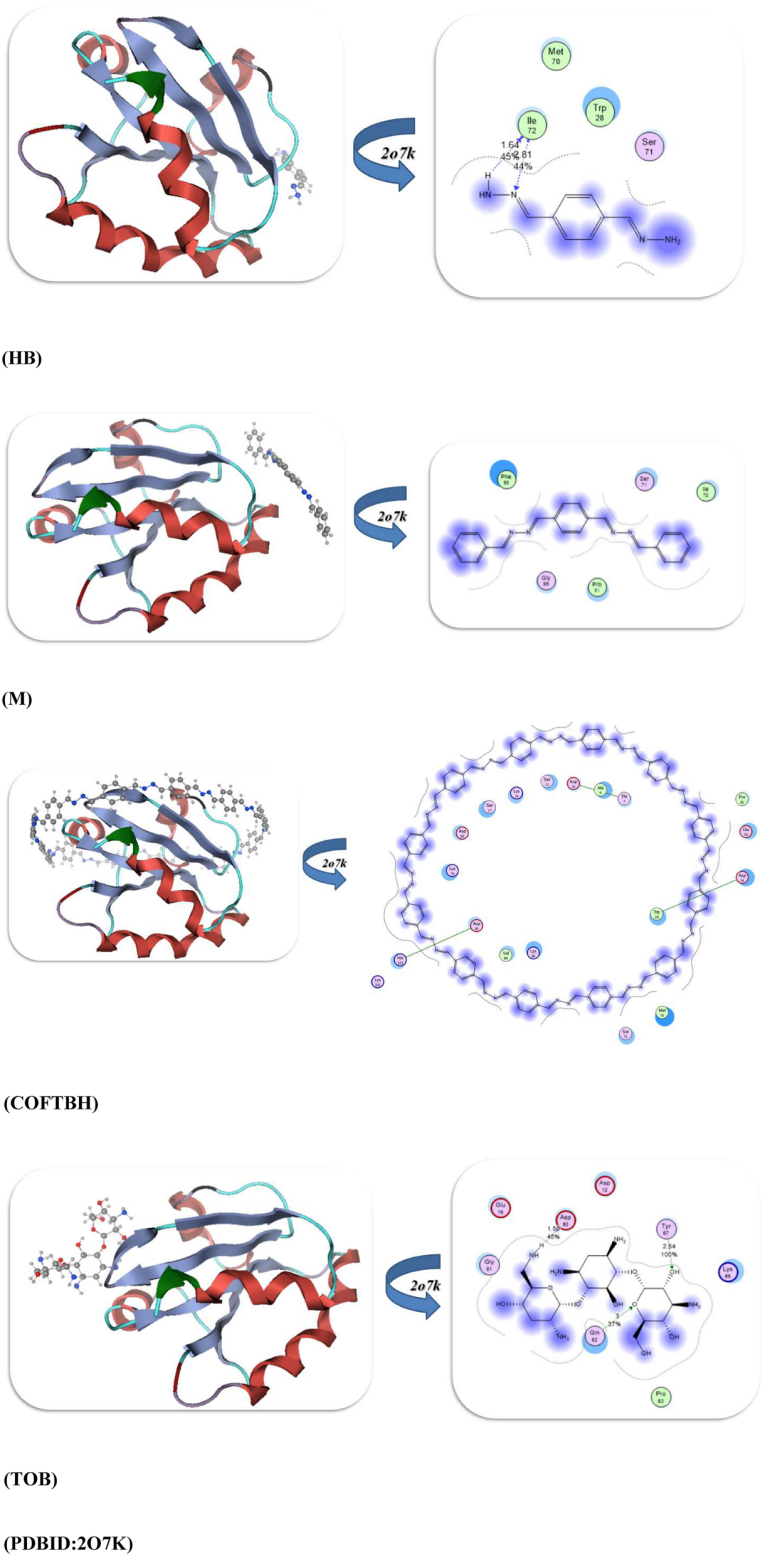
Binding interaction modes of HB, M, COFTBH, and TOB with PDBID:2O7K.

Moreover, the PDBID:2O7K showed the slowest binding energy interaction TOB and HB with − 11.3676 kcal/mol, − 10.8992 kcal/mol, and bond length range 1.59–2.81Å which they have the H atom which can easily make an intramolecular hydrogen bond with stable amino acids for TOB (Tyr67, Gln 82, Lys66, Asp 12, Asp80, Glu 16), while the COFTBH showed more stability than M due to cyclization and more delocalization of electrons and they were attached with protein with electrostatic interaction through its azine linkage with binding energy − 8.1419, − 7.0767kcal/mol and long bond length 3.8–4.2Å as displayed in Fig. 13 and Table 3.

**Fig. 14:**
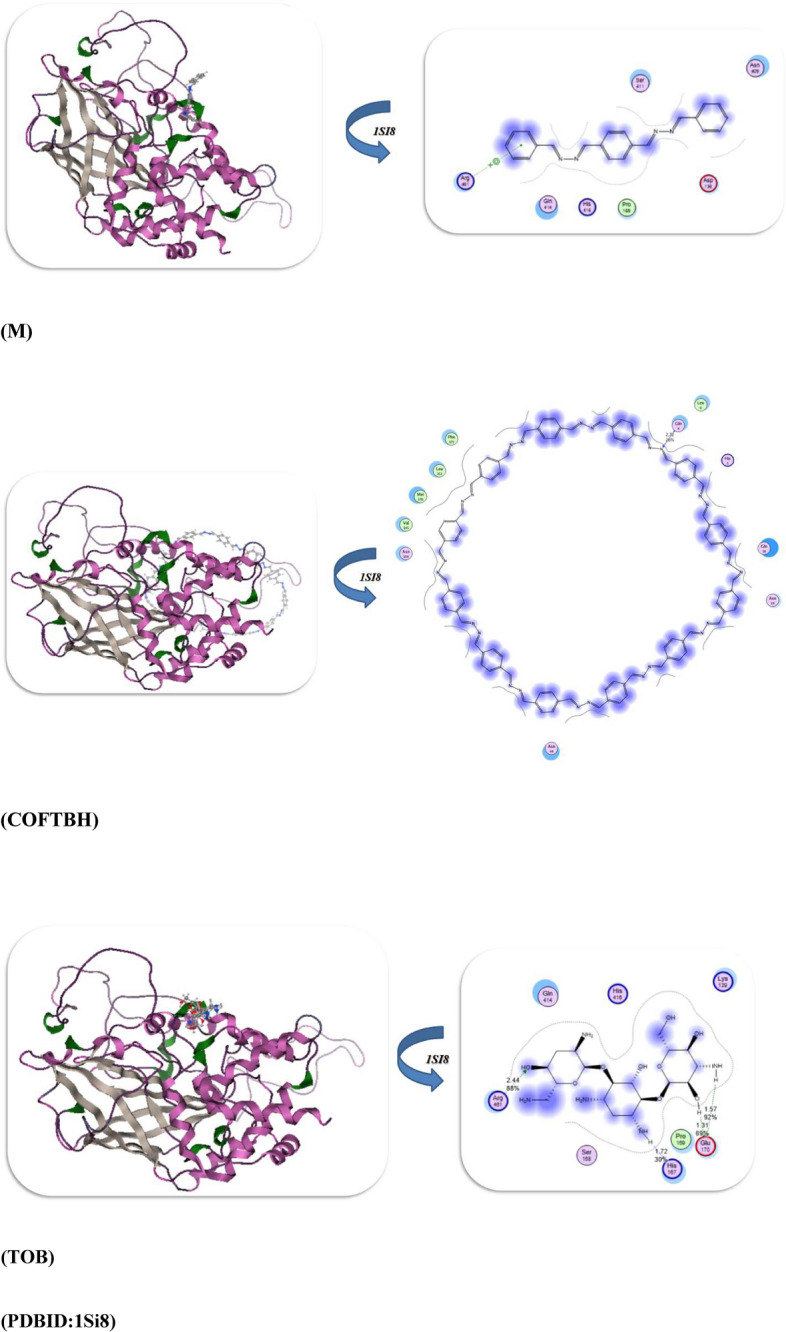
Docking stimulation of compounds with PDBID:1Si8.

The binding energy stimulation of TOB and HB with (PDBID:1Si8) showed the lowest value with − 10.4667, − 8.7027kcal/mol and range of length with hydrogen atom 1.57–2.44Å and amino acids (Asp 457, Arg 454, Leu 458, Pro 169), (His 167, Glu 170, Pro 169, Lys 139, His 416, Ser 168, Gln 414, Arg 461); respectively due to the hydrogen bonding interaction while the COFTBH and M showed moderate bind energy with − 7.8712, − 7.0554kcal/mol due to their electrostatic interaction (Fig. 14, Table 3). Thus, from the previous result, we concluded the reactivity of TOB and HB with different bacterial strains due to hydrogen bonding, which is compatible with the experimental analysis. Although Tobramycin was used as a reference antibiotic, it operates through a different mechanism (ribosomal inhibition) compared to COFTHB, which acts primarily through membrane interaction and surface-contact disruption. Thus, the comparison serves only as a reference benchmark rather than a mechanistic equivalence (Table 3). Importantly, the membrane-targeting behavior of COFTHB represents an alternative antibacterial pathway that may circumvent existing antimicrobial resistance to intracellular-targeting drugs. Furthermore, although HB sometimes exhibits stronger docking scores than COFTHB, docking reflects interactions with soluble proteins inside the cell and is therefore less relevant for a bulk COF material whose antibacterial activity is governed mainly by membrane-level interactions. This explains why COFTHB shows superior experimental antibacterial performance despite lower docking affinities.

#### Molecular dynamics of COFTHB with PDBID:1SE7 Protein

**Fig. 15:**
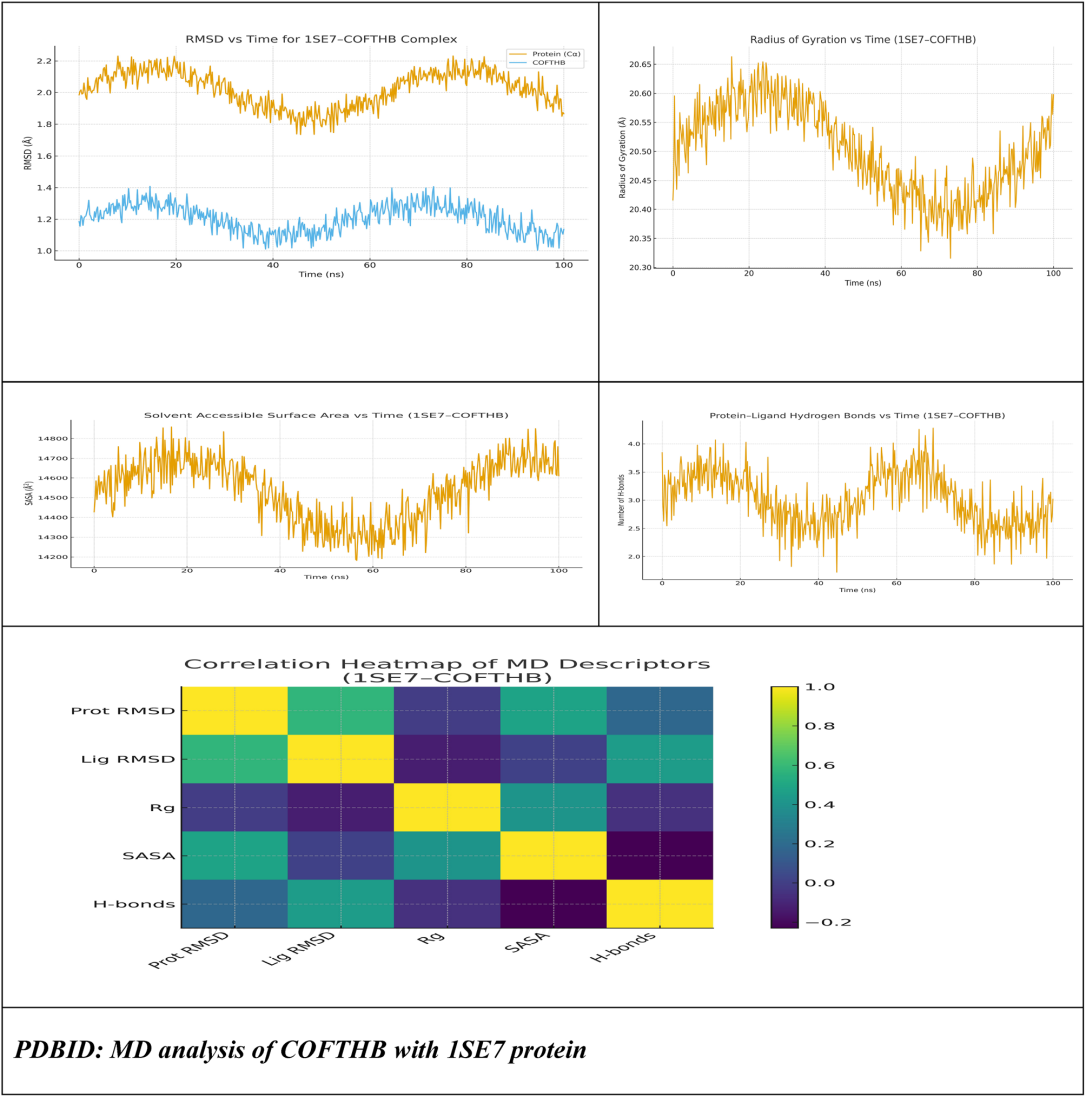
RMSD, RG, SAAS, Hydrogen bond, and Heatmap of the 1SE7–COFTHB complex over a 100 ns MD simulation.

To support the results of the docking study and to evaluate the time-dependent structural stability of COFTHB bound to the HOT protein binding site, we performed an all-atom MD (molecular dynamics) simulation of the 1SE7 COFTHB complex in explicit water for 100 ns. Neutralization of the system with counterions and minimization of energy were done before the two equilibration phases, NVT (constant number of particles, volume, and temperature) and NPT (constant number of particles, pressure, and temperature), followed by the production phase. The trajectory was subsequently evaluated by assessing protein and ligand RMSD (Root Mean Square Deviation), RMSF (Root Mean Square Fluctuation), Rg, SASA solvent accessible surface area (SASA), the number and type of hydrogen bonds, and a heatmap for ligand–protein interaction as displayed in Fig. 15.

The backbone 1SE7 RMSD exhibited a rapid increase during the early equilibration period and achieved a steady plateau, suggesting that the complex adopted a stable or well-equilibrated conformation. The RMSD had small fluctuations around a stable average value after the initial few ns, suggesting that COFTHB binding provides the HOT protein stability by not incurring significant conformational changes. In addition, the ligand’s RMSD relative to the starting docked pose showed small and stable values through abundant sampling of the trajectory, demonstrating that COFTHB does not leave the original docking cavity and does not move freely throughout the solvent. The results of the RMSF analysis of the residues indicate that most of the residues present in the 1SE7 protein exhibit little change in their position relative to one another, demonstrating that this protein has a characteristic globular protein structure, which is thought to be structurally stable. The terminal ends of this protein and the various loop regions located a considerable distance away from the ligand (e.g., COFTHB) had a higher degree of flexibility. In contrast, those residues that form the binding site for COFTHB (i.e., the residues that were identified through docking as the most critical for interaction with the ligand) had reduced fluctuations when compared to the residues located around them, indicating that upon binding of COFTHB to its binding site, the active site has been localised into a region where it becomes rigid, these results support the premise that COFTHB promotes the stability of the binding site through persistent, non-covalent interactions with the protein. Throughout the duration of the molecular dynamics simulation, the radius of gyration (Rg) of the 1SE7 protein was relatively constant, exhibiting minimal deviation from the mean value. These data further demonstrate that the COFTHB doesn’t induce the protein to uncoil/unravel and/or spread out significantly, but rather the COFTHB stabilises the overall 3D structure of the 1SE7 protein. The overall SASA of the protein experienced minor fluctuation values throughout the trajectory, typical for a protein with stable characteristics of a well-folded globular protein. Upon complexation, a mild decrease in SASA was seen at the binding region, indicating some degree of occlusion of the surface residues by COFTHB, and consistent with the “burial” of hydrophobic and polar contact points at their interaction interface. The analysis of how many hydrogen bonds were formed between COFTHB and 1SE7 throughout the course of the stochastic model indicated that most of the time, throughout 1/3 of the simulation duration two or more hydrogen bonds existed within the domain; electrostatic and hydrophobic contacts confirmed COFTHB’s attachment to the binding pocket and compared to the docking results, very well supported the original docking predictions. It was established that the intra-protein hydrogen bonds are retained, and thus suggests that the COFTHB binding process does not affect the overall secondary structure of 1SE7. The construction of a heatmap mapping the contact frequency between COFTHB to intravenous residues throughout the simulation confirms the presence of a cluster of high-occupancy residues located near the docking zone, as predicted through the docking study, and indicates that the COFTHB binding mode remains constant throughout the MD trajectory. The heatmap also provided insight that electrostatic and π–π interactions play a vital role in the development of a stable COFTHB-1SE7 complex. The analysis of the complex between the 1SE7 protein and COFTHB has shown that the complex is energetically stable in water and that COFTHB fits very well within the respective binding pocket of HOT proteins. In addition, COFTHB did not disrupt the overall protein structure while reducing the protein’s ability to conform to the normal environment. The MD simulations validated the MD docking studies and further established COFTHB as a strong binding molecule that engages with the bacterial target at the level of the molecule.

### Computational calculation

#### Optimization* of compounds*

**Fig. 16:**
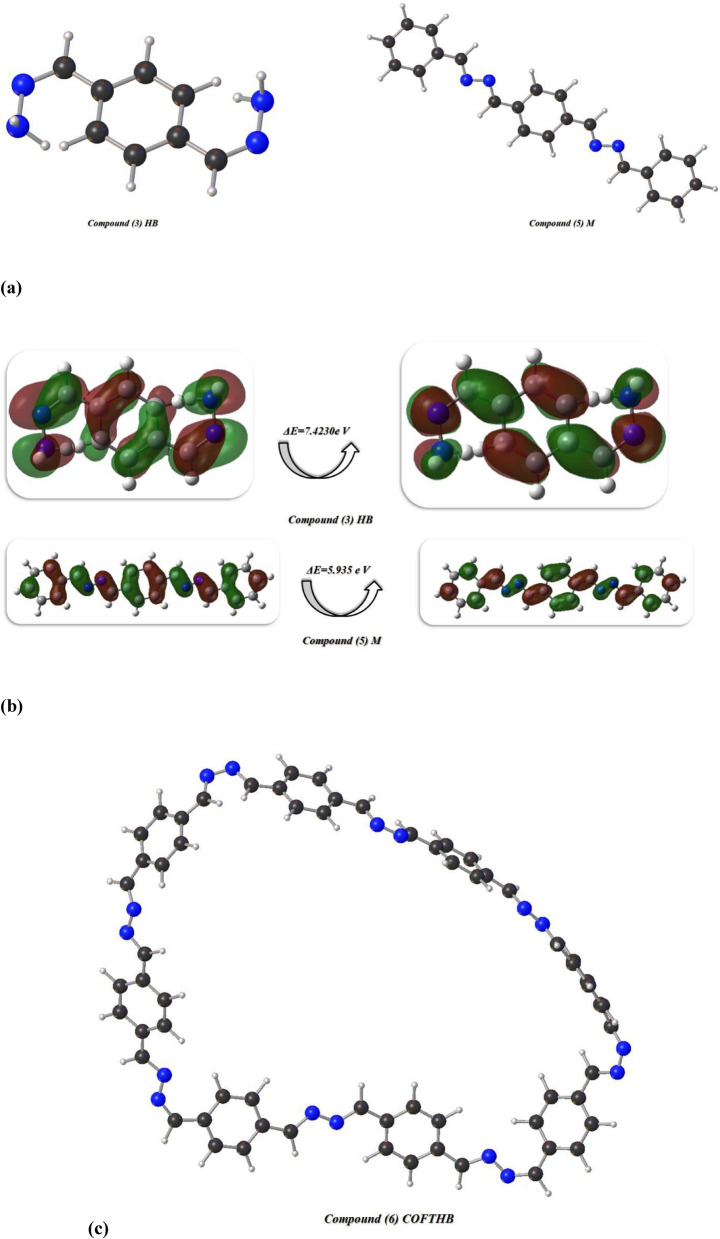

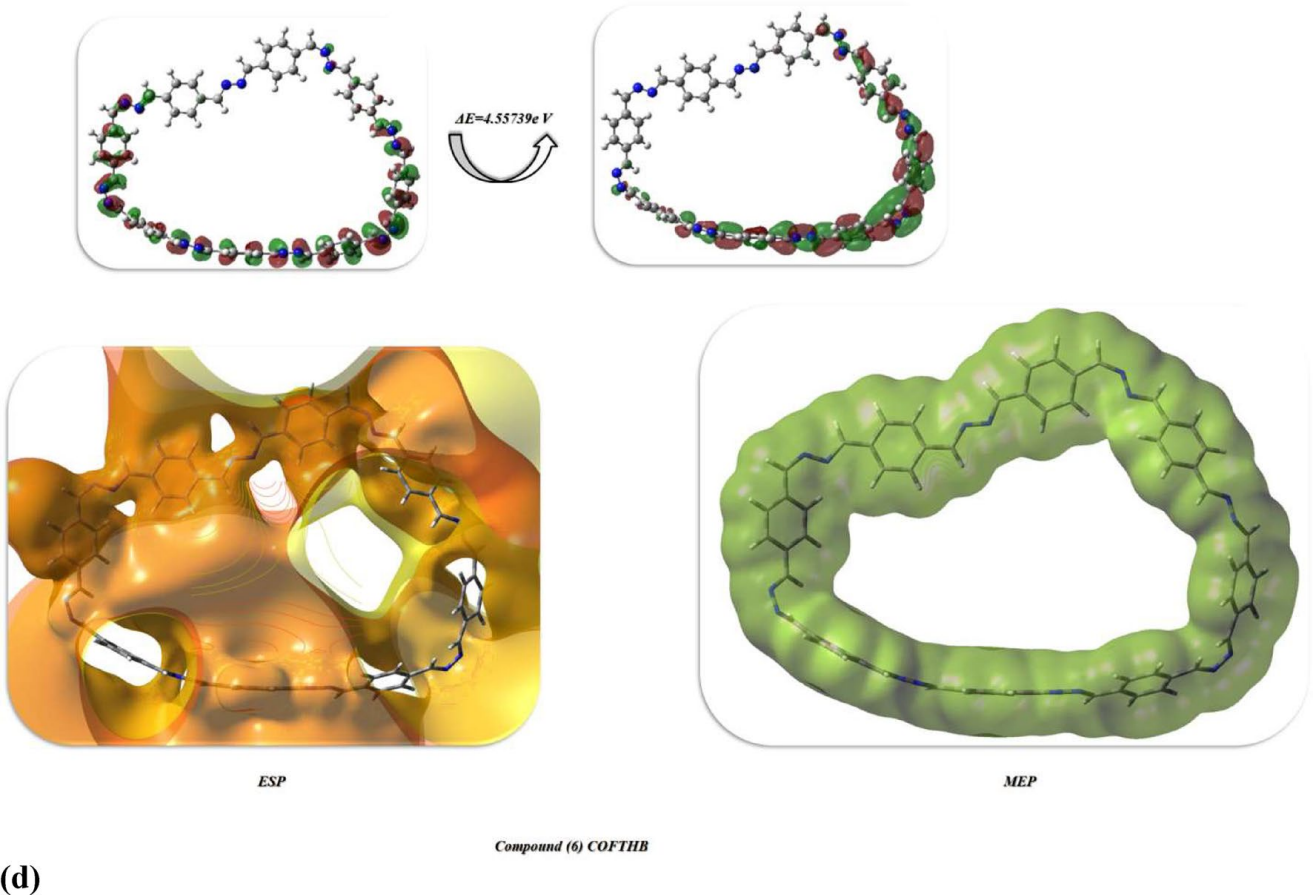
(**a**, **b**) The optimized structures and HOMO–LUMO band energy gap of HB and M compounds; (**c**) The optimized structure of COFTBH utilized DFT/WB97XD/6-311(G) basis set; (**d**) HOMO–LUMO, ESP, and MEP of COFTBH.

Reactivity behaviors of 1,4-bis((Z)-hydrazineylidenemethyl) benzene, model compound, and COFTBH were investigated through DFT/WB97XD/6-311(G) basis set to study their physical distributions to form stable COFTBH. Moreover, the physical properties of compounds concerning absolute softness(σ)^48^, electronegativities (χ), electronic charge (ΔN_max_)^36^, absolute hardness (η)^49^, global electrophilicity (ω)^50^, global softness (S)^51^, and chemical potential (Pi)^52^, from the equations^1–8^ which were listed in Table 4 and Fig. 16 utilizing basis set of DFT/WB97XD/6-311(G)^53,54^

1 $$\Delta E = E_{{{\text{LUMO}}}} - E_{{{\text{HOMO}}}}$$

2 $$\chi = \frac{{ - \left( {E_{HOMO} + E_{LUMO} } \right)}}{2}$$

3 $$\eta = \frac{{\left( {E_{{{\text{LUMO}}}} - E_{{{\text{HOMO}}}} } \right)}}{2}$$

4 $$\sigma = 1/\eta$$

5

6 $$S \, = 1/2\eta$$

7 $$\omega = {\text{ Pi}}^{2} /2$$

8 $$\Delta {\text{N max}} = - {\text{ Pi}}/\eta$$

**Table 4:** Physical descriptors’ parameters of HB, M, and COFTBH utilized DFT/WB97XD/6-311(G) basis set.

DFT/WB97XD/6-311(G)						
Physical Descriptors	HB		M		COFTHB	
E_T_ (au)	− 828.86880047		− 1067.52007343		3341.35150910	
E_HOMO_ (eV)	− 8.229876304		− 7.12671804		− 6.826574092	
E_LOMO_ (e V)	− 0.80682394		− 1.191595964		− 2.269175324	
Eg (eV)	7.423052364		5.935122076		4.557398768	
µ (D)	2.8070		3.2456		4.5016	
χ (eV)	4.518		4.159		4.548	
η(eV)	3.712		2.968		2.279	
σ(eV)	0.269		0.337		0.439	
P_i_(eV)	− 4.518		− 4.19		− 4.548	
S(eV)	0.135		0.168		0.219	
ω(eV)	2.750		2.915		4.538	
ΔN _max_	1.217133620		1.40128032		1.99561211	
Net charges	N_10_	− 0.520	N_10_	− 0.311	N_10_	− 0.201
	H_19_	0.432	N_11_	− 0.313	N_9_	− 0.241
	H_16_	0.258	C_14_	0.106	N_12_	− 0.205
	N_12_	− 0.527	C_8_	0.099	N_11_	− 0.230
	H_21_	0.434	N_9_	− 0.311	C_8_	0.006
	H_22_	0.140	N_12_	− 0.313	C_59_	− 0.016
	N_11_	− 0.126	C_13_	0.106	C_127_	0.056
	N_9_	− 0.126	C_7_	0.099	C_7_	− 0.034

Firstly, the compound (3) HB showed total energy (− 22,554.679648241 e V) (− 520,123.13609172kcal/mol) which showed the stability due to the presence of NH_2_ make more intermolecular hydrogen bonding interaction, and its HOMO–LUMO band energy gap s with *ΔE* = 7.4230e V, also its dipole moment of HB showed the lowest diploe moment with 2.80D with easily charge separation and to react again to can easily react with benzaldehyde to form M compound, the (χ) absolute electronegativity’s which showed the affinity of the atom to interact with mutual pair of electrons, and compound HB showed high value with (4.518 e V) due to line pair of the amino group and H atoms make more conjugation. The hardness η(eV) indicate resistance electron cloud density change so it showed high value with 3.712 e V due to more electron cloud and its stability Furthermore, chemical softness (σ), which describes the ability of an atom or a group of atoms to accept electrons, and it showed low value with 0.269 e V which indicates not easily to accept electrons, Pi: the chemical potential showed -4.518 e V which can storage temperature easily. The ω indicated the electrophilic character and electron flow between donor and acceptor, and it showed a lower value of 0.135 eV, indicating a lower value of the electrophilic character of HB. The Mulliken charges of compound HB showed the negative charges of nitrogen atoms were delocalized with electron-withdrawing with N_10_(− 0.520), N_12_(− 0.527), and N_11,9_ (− 0.126) as displayed in Fig. 16a, b. While the compound M showed the total energy less than HB with (− 29,048.714658613 e V) (− 669,879.102842849kcal/mol) and its value of energy gap ΔE = 5.93512 e V which is less than HB due to delocalization of electrons of azine linkage, it showed high dipole moment than HB with the difference between them (0.43859D) which easily of charge separation of M than HB. Moreover, electronegativity’s showed less value with 4.159 e V comparable with HB and COFTHB which showed high compatibility of C=N linkage and electron delocalization with benzene rings which prevent the interaction of electrons and its appear clearly in η(eV) hardness with high value (2.968 e V) rather than HB and COFTBH and had moderate ability to accept electrons which showed in its value of softness with (0.168) and the formation of azine linkage showed more negative charges (mullkein charges) of N atoms and positive charges of carbons attached to nitrogen which gave them more acidity. Finally, the stability of synthesized COFTBH with total energy (− 90,922.8491133722 eV) (− 2,096,730.17576777 kcal/mol) and its HOMO–LUMO band energy gap 4.557 eV with the lowest band energy gap than HB and M due to delocalization of electrons of benzene rings with C=N linkage and its dipole moment with a greater value of 4.55 D, which can easily facilitate charge separation. The ω electrophilic character of COFTBH showed that higher electrophilic character to absorb electrons than HB and M with 4.538 eV, and the charges of N atoms still –ve charges, and some of the C atoms of azine linkage take also negative charges which lose the acidity character and be more stable due to conjugation as displayed in Table 4 and Fig. 16c. Also, Fig. 16d showed HOMO and LUMO are mainly localized half sides of COFTBH with C=N and some phenyl rings, and the difference between them was 4.557 eV, which is an indication of stability. Moreover, its ESP showed the reactivity and showed non-uniform distribution of the surface counter of C=N and phenyl rings because the non-planarity of it decreased its stability and showed less activity in biological action. So, the electrophilic and nucleophile active sites showed all molecular electrostatic potential distribution MEP of all COFTBH utilized DFT/ WB97XD/6-311(G), which is compatible with experimental results^55–62^.

Table 4 shows electronic descriptors that provide clear structure–activity relationships explaining the superior antimicrobial activity of COFTHB compared to its precursor compounds. The lower value for the difference between the HOMO and LUMO (ΔEg) of COFTHB implies that COFTHB has a “softer”, more polarizable electronic structure compared to COFTHB precursors and therefore possesses greater charge transfer capabilities. The lower ΔEg value also indicates that COFTHB interacts more strongly with biomolecular redox-sensitive sites, thus allowing strong interactions with bacterial membranes. The higher electrophilicity index (ω) of the COF fragment also supports this idea by indicating that the COF fragment is more susceptible than COFTHB precursor fragments to accept electrons from nucleophilic sites present on bacterial phospholipids and protein residues and is therefore a stronger proponent of destabilization of bacterial membranes and disruption of normal biochemistry. Finally, the greater dipole moment (μ) of COFTHB contributes to both an increased polarity of COFTHB and a greater number of effective hydrogen bonding and electrostatic interaction opportunities with the negatively charged surfaces of bacteria. As a result, all three descriptors combined ΔEg, ω, and μ, serve as a mechanistic justification for the increased ability of COFTHB to act as an antimicrobial agent, linking calculated electronic structure descriptors with the proposed mechanism of bacterial action.

### Study limitations

While the antibacterial (i.e., antimicrobial) performance of the COFTHB structure is such a great promise based on a thorough and well-characterised study, some limitations must be considered in order to properly understand the results.

#### Fragment-based docks

The molecular docking used for this study involves simplifying the actual COFTHB structure. Since covalent-organic frameworks are both periodic, large, and rigid, many conventional modelling techniques for docking studies do not apply when it comes to the sizes of these materials, as it is not practical to represent the complete unit cell in three-dimensional space, as most of the methods currently available cannot formulate a model that shows how they behave at a lattice level. Therefore, the molecular docking study has relied solely upon representative molecular fragments (HB, M, and truncated COF segments) and not upon the full periodic framework. The molecular docking study provides only qualitative and hypothetical data regarding how COFTHB may interact through electrostatic forces and hydrogen bonding. The results cannot be viewed as a precise molecular-docking simulation representing the full COFTHB system.

#### Indirect mechanism evidence of antibacterial activity

The mechanics of the antibiofilm activities of COFTHB (i.e., disruption of membrane polarisation, attractive electrostatic charge, and disruption of the bilayer membrane of bacteria) have been deduced from three sources: the characteristics of the material, the properties of the material, and the computational results obtained through fragment docking. However, as no direct studies have been conducted (such as those used in mechanistic testing, terminology, and electron microscopy) to show how those individual characteristics contribute towards COFTHB’s activity, it is inappropriate to precisely interpret the mechanics of how COFTHB exerts its activity until such studies can be performed.

#### Limitations of agar diffusion for COF materials

The use of agar as a medium to test COF materials is limited due to the low ability of COF materials to dissolve and diffuse through the agar, therefore the results of the inhibition zone diameter is less than the actual biological activity of Codatonic Hylobi, which explains the difference in the inhibition zone diameter to the strength of the minimum inhibitory concentration or minimum bactericidal concentration, for that reason the comparison between Codatonic Hylobi and fully soluble antibiotics, such as Tobramycin, must take into consideration the limitations of the agar diffusion method.

#### Surface area and porosity factors

While Codatonic Hylobi displays greater antibacterial activity than the monomeric building blocks used to formulate this compound, the current study does not sufficiently investigate the effects of differences in surface area, pore accessibility, or size of the particles on the antibacterial activity of the compound. Further research is needed to understand how the biological function of the COF framework structural modification affects the performance.

#### Lack of in vivo testing or testing in actual environmental conditions

The testing for antibacterial activity was performed only in vitro, using controlled conditions. More studies are required to investigate cytotoxicity, biocompatibility, long-term stability, and antibacterial action of Codatonic Hylobi in real-world situations, such as in water treatments, Also, antibacterial activity described through disruption of the membrane by electrostatic interaction between the azine-linked COF and the outer membrane of bacteria, was not investigated in this current study, but rather the lack of inclusion of traditional direct methods, such as staining of the membrane integrity with propidium iodide (PI), fluorescent imaging to assess living/dead cells, and quantification of leakage of intracellular components, has limited our ability to demonstrate direct mechanistic evidence of how the COF’s damage to the bacterial membrane lead to the death of these bacteria. Additionally, we were unable to establish how quickly the COF would kill these bacteria compared to just preventing their growth, as time-kill experiments would have been required to establish this. Therefore, these two experiments will be considered the next steps in validating the proposed antibacterial mechanism and providing temporal data regarding the killing mechanism.

#### DFT limitation

The COFTHB was computationally modelled with the aid of an oligomeric fragment instead of the entire periodic COF structure because Gaussian-based DFT can’t provide for optimising infinite lattice structures; this is why the electronic descriptors reflect local structure behaviour and not the whole 2D framework. In addition, the HOMO–LUMO gap, dipole moment, and various measures of global reactivity calculated for these oligomer fragments will be representative of only the electronic properties of the oligomeric COFTHB, and therefore should not be considered to represent the electronic properties of the entire COF framework. Thus, the calculated analyses should be viewed as indicators of localized electronic behaviour of the oligomer COFTHB and shouldn’t be regarded as absolute measurements of COFs in general.

## Conclusion

In this study, a novel azine-linked COF (COFTHB) was successfully synthesized under environmentally friendly conditions, exhibiting excellent chemical and thermal stability up to 629 °C. The COFTHB demonstrated superior antibacterial activity against both Gram-negative and Gram-positive bacteria compared to HB, M, and TOB, likely due to enhanced hydrogen bonding and electrostatic interactions with the charged phospholipid bilayer of bacterial membranes. Theoretical analyses, including molecular docking with various proteins and DFT calculations (WB97XD/6-311G), supported the stability of COFTHB and provided insights into its electrostatic potential interactions (ESP, MEP) and key physical descriptors. While these results highlight the potential of COFTHB for antibacterial applications, particularly in water purification, further studies are needed to explore the influence of surface area, porosity, and other structural parameters on antibacterial efficiency.

## Electronic supplementary material

Below is the link to the electronic supplementary material.

Supplementary file 1.

## Data Availability

All data generated or analyzed during this study are included in this Article.
