# Supplementary material for: Green synthesis of 2D azine-linked covalent organic framework with antibacterial activity correlated by molecular docking study and computational calculations
Source: Sci Rep. 2026 Jan 10;16:1335. doi: 10.1038/s41598-025-32900-3 (PMC12796362; doi:10.1038/s41598-025-32900-3)
Supplement: Supplementary file 1 [file 41598_2025_32900_MOESM1_ESM.docx]

**Supporting Information for**

**Green Synthesis of** **2D Azine-Linked Covalent Organic Framework with Antibacterial Activity Correlated by Molecular Docking Study** **and Computational Calculations**

**Eman Abdelnasser^a,⁎^, Asmaa M. Fahim^b^,** **Daniel T. Oyekunle^c^,** **Abd El-Motaleb Mosad Ramadan^a^, Ahmed I. Khodair^a,⁎^**

*^a^* Chemistry Department, Faculty of Science, Kafrelsheikh University, El-Geish Street, P.O. Box 33516, Kafrelsheikh, Egypt.

*^b^*Green Chemistry Department, National Research Center Dokki, P.O. Box 12622 Cairo, Egypt

*^c^* Institute of Sustainable Energy, Universiti Tenaga Nasional (UNITEN), 4300 Kajang Selangor,

Malaysia

**Corresponding Author:** * E-mail: [eman_ahmed2014@sci.kfs.edu.eg](mailto:eman_ahmed2014@sci.kfs.edu.eg); [khodair2020@yahoo.com](mailto:aik@yahoo.com)

**Section 1. Materials and Methods**

The terephthalaldehyde, dioxane, ethanol, acetic acid, benzaldehyde, and hydrazine hydrate were obtained from Aldrich. The organic solvents were distilled over appropriate drying reagents under nitrogen. Deuterated solvents for NMR measurement were obtained from Aldrich.^1^H and ^13^ C spectra were recorded on a variation GEM-400 NMR spectrometer, where tetramethylsilane as internal standard and chemical shifts are expressed as δ ppm. The infrared spectra were recorded

from 400 to 4000 cm^-1^ Thermo Fisher Scientific (Waltham, MA) spectrometer by using KBr pellets. X-ray diffraction (XRD) patterns were recorded on a (Pertpro,Cu k_a1_radiation, k= 1.5404 A ^̊^, 45 kV, 40 mA, USA) diffractometer. The taken range at 2*θ* = 4.0° to 80° with 0.02° increment at 25 °C. UV/Vis spectra have been carried out on a Perkin Elmer Lambda 950 spectrophotometer within the wavelength range 200–600 nm. Thermogravimetric analysis (TGA) was performed on a Shimadzu thermogravimetric analyzer TGA-50, thermogravimeter by measuring the weight loss while heating at a rate of 10 °C min−1 from 25 to 1000 °C under nitrogen. Nitrogen sorption isotherms were measured at 77 K with a JW-BK 132F analyzer. The micrographs of the prepared materials were obtained by using scanning electron microscopy (SEM) (JOEL, JSM, IT100). Samples were mounted onto a graphite substrate and coated by gold to record micrographs in the range of 15–30 keV and the working distance was approximately 10–15 mm. Before measurement, the samples were degassed in vacuum at 120 °C for more than 10 h. The Brunauer-Emmett-Teller (BET) method was utilized to calculate the specific surface areas and pore volume, BJH method was applied for the estimation of pore size distribution.

**Section 2. Syntheses and characterization**

**Preparation of COFTHB for NMR**

COF is dispersed in DMSO-d₆, and gentle heating was applied (including brief microwave-assisted heating).

**Synthesis of model compound.**

Model compound was synthesized by condensation of Benzaldehyde and 1,4-bis(Z) hydrazonnmethyl benzene in the presence of the acetic acid as catalyst in 100 mL of ethanol under reflux then the ppt was formed and washed with MeOH, dried under vacuum to afford model compound as bright yellow powder in 91 % yield. ^1^H NMR (DMSO, 300 MHz): *δ* (ppm) 7.49 (8H, m, Ar-H), 8.03 (4H, s, Ar-H), 8.79 (4H, s, CH). IR (KBr, ν; cm^–1^): (-C=N) band at 1613 cm^-1^.

**Characterization of 1,4-bis(Z)-hydrazonnmethyl benzene**

The successful preparation of 1,4-bis(Z)-hydrazonnmethyl benzene was approved by FT-IR (Fourier transform infrared) spectroscopy. The FT-IR spectrum of 1,4-bis(Z)-hydrazonnmethyl benzene exhibited the absence of stretching bands of carbonyl C=O (1689 cm^-1^) of terephthaldehyde and the amine group of peaks of 1,4-bis(Z)-hydrazonnmethyl benzene (3349, 3187 cm^-1^) and formation of imine functional groups C=N at (1607 cm^-1^). UV-V spectrum 307 and 360 nm; ^1^HNMR of 1,4-bis(Z)-hydrazonnmethyl benzene (400 MHz, DMSO-d_6_, δ, ppm): 6.57 (4H, br. s, NH_2_), 7.44 (4H, s, Ar-H), 7.80 (2H, s, CH); ^13^C-NMR (100 MHz, DMSO-d_6_, δ, ppm): 125.58 (4c-Ar), 135.66 (2 C-CH=), 140.64 (2CH=).

**Characterization** **model compound**

The model compound was prepared and showed the -C=N band at 1613 cm^-1^. ^1^HNMR of model compound (400 MHz, DMSO-d_6_, δ, ppm): 7.49 (8H, m, Ar-H), 8.03 (4H, s, Ar-H), 8.79 (4H, s, CH).

**Characterization of COFTHB**

FT-IR spectroscopy revealed the successful synthesis of the novel COFs. The FT-IR spectrum of COF did not reveal the typical carbonyl stretching bands C=O (1711cm^-1^) of terephthaldehyde and amine group of peaks of 1,4-bis(Z)-hydrazonnmethyl benzene and

**Fig. S1.** FTIR of model compound


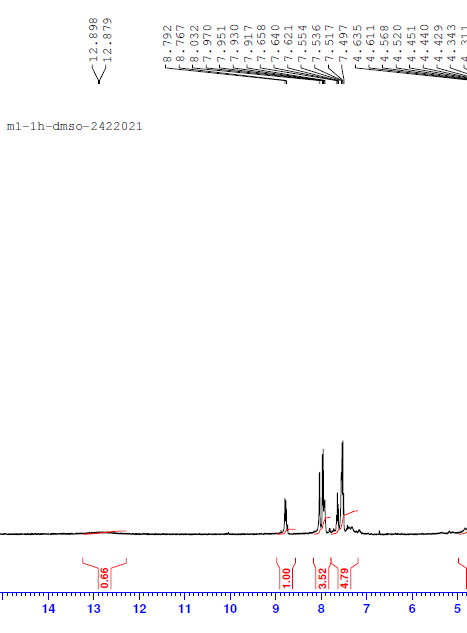


**Fig.** **S2.** ^1^H-NMR of model compound


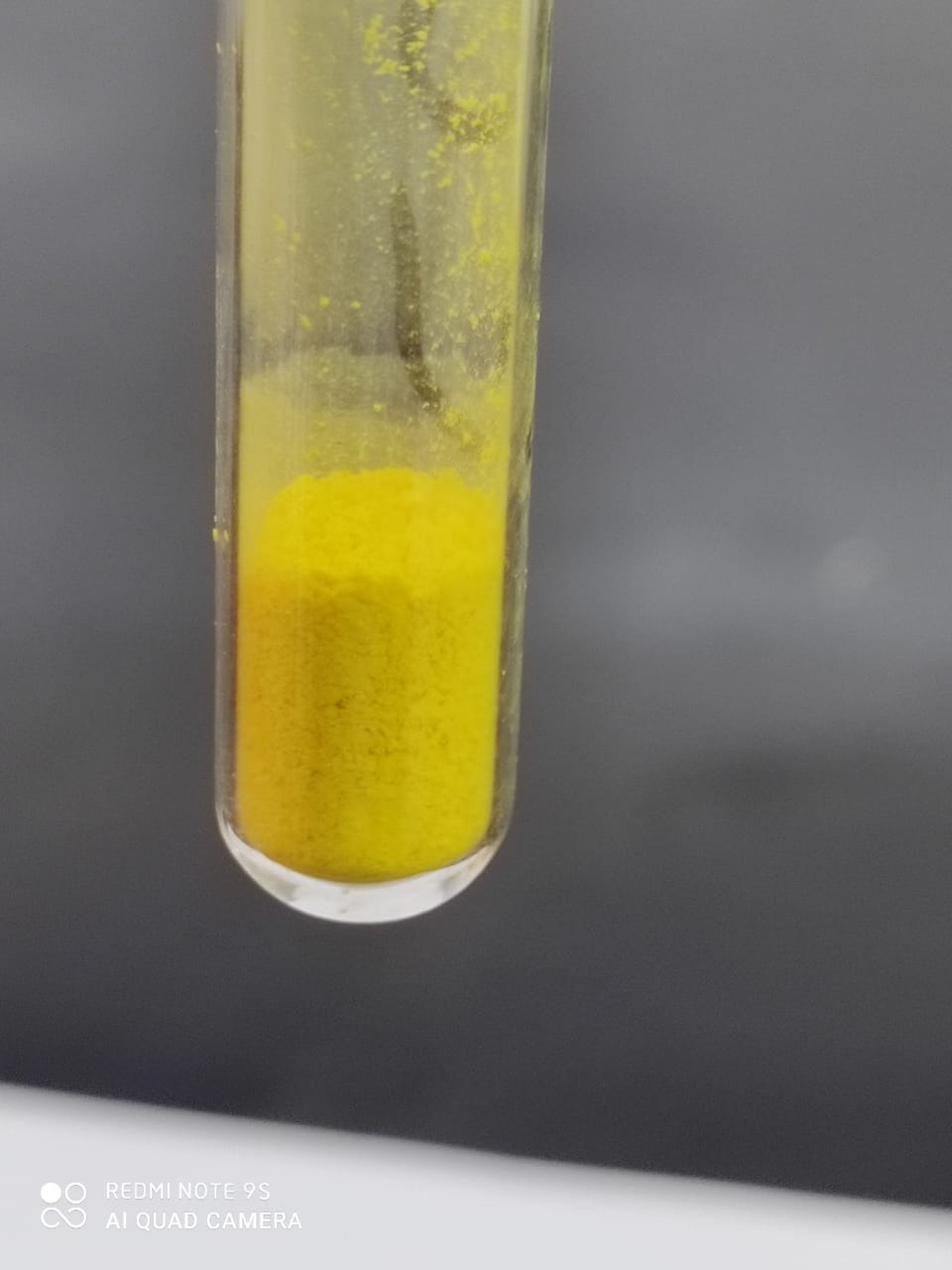


**Fig. S3.** Supporting photo of model compound


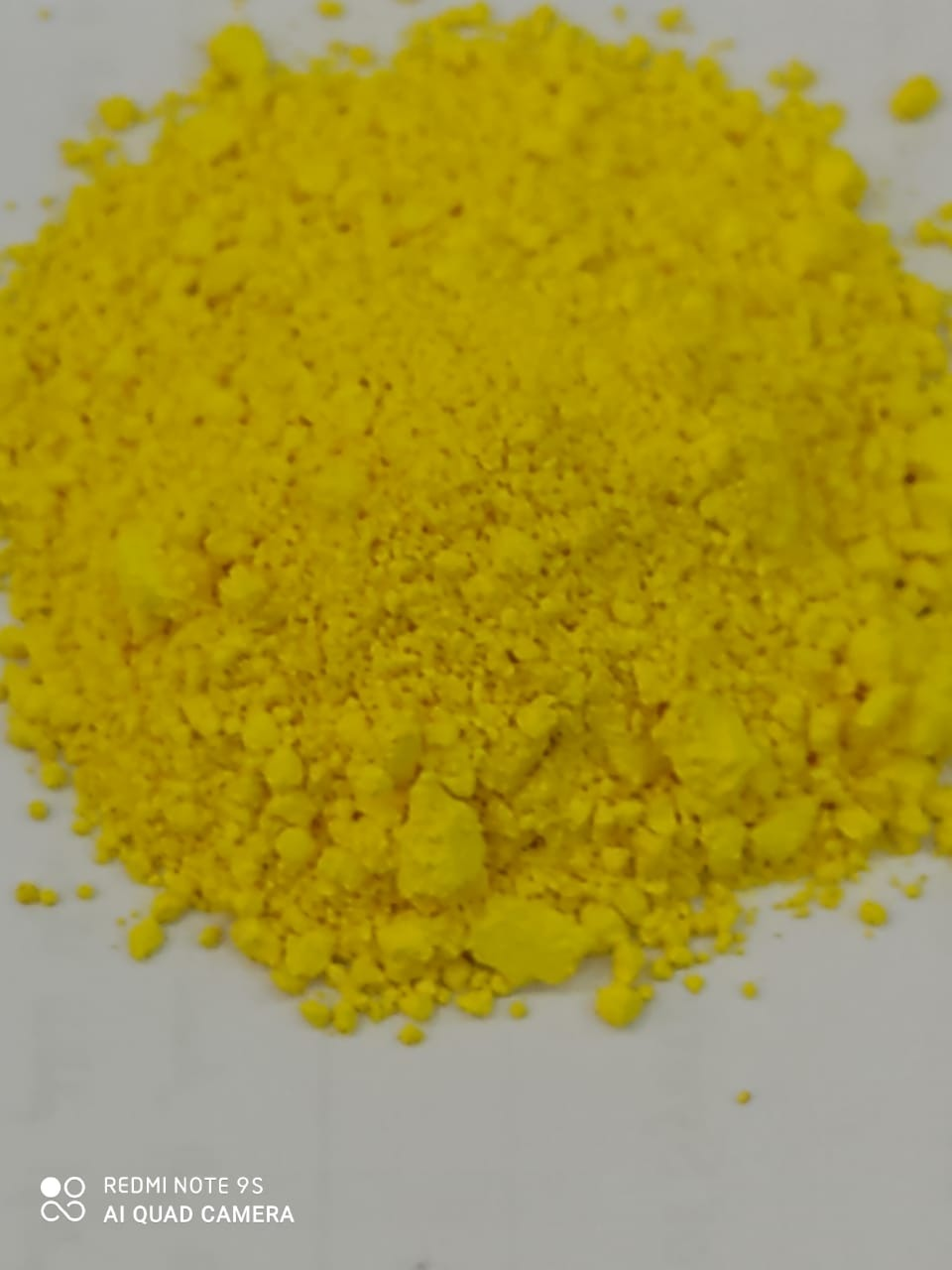


**Fig. S4.** Supporting photo of COFTHB


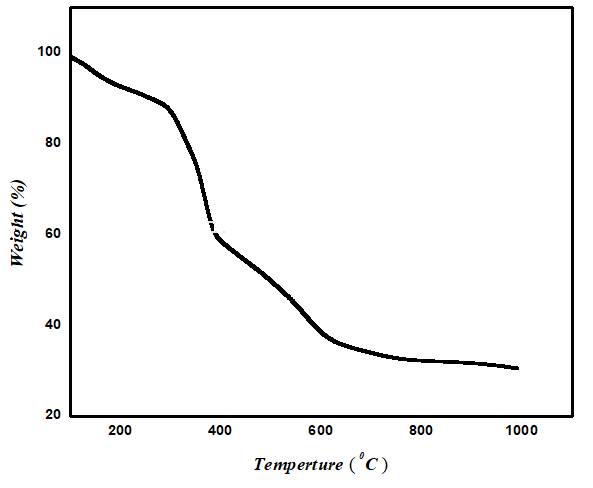


**Fig. S5.** TGA of COFTHB


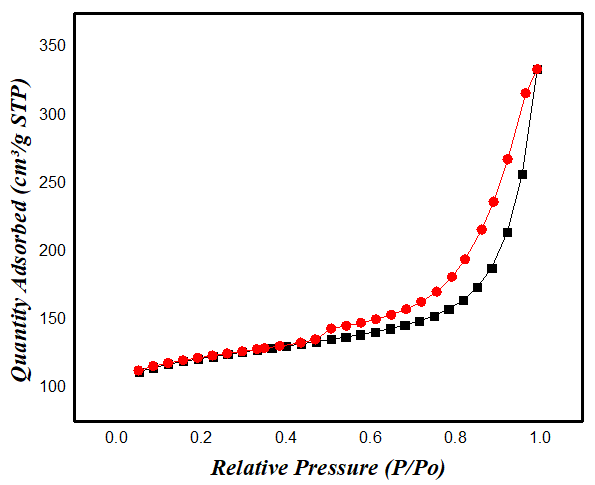


**Fig. S6.** N_2_ adsorption-desorption isotherm of COFTHB

**Section 2. Structural simulation and Powder X-Ray Diffraction Analysis**

The Pawley refinement of the experimental PXRD was conducted by the Reflux module in the Material Studio 7.0, resulting in the cell parameters a = b = 14.480 Å and c = 3.610 Å with Rp = 5.59% and Rwp = 8.18%. Before the simulation, the structure was firstly optimized in Gaussian 09 package by semiempirical calculations at PM3 level. The simulation of the four possible structures was carried out in Accelrys Material Studio 7.0 software package. The stimulated PXRD patterns were determined by the Reflex module. And the unit cell was optimized by Forcite module

under molecular mechanics calculation using COMPASS II as the forcefield to give the relative total energy. The initial lattice parameters and fractional atomic coordinates of AA.

**Table S1. The Space Groups and Cell Parameters of the two possible structures of** **COFTBH**

| **Structure** | **Crystal System** | **Space Group** | **Cell Parameters** |
| --- | --- | --- | --- |
| AA | Orthorhombic | PBCA | a=b=37 Å, c= 8.9  α=β= γ= 90º |
| AB | Orthorhombic | Fddd | a=b=37 Å, c= 12.36  α=β= γ= 90º |

**Section 3. Antibacterial activity**

The groups of bacteria Escherichia coli, Pseudomonas aeuroguinsa (Gram-negative bacteria) and staphylococcus aureus, Enterococcus faecalis (Gram-positive bacteria) were used to investigate the antibacterial activities of the thienopyridines and their nanocomposites. Cut plug method, recorded by Pridham et al. 1956 [1], was used as follows: Freshly prepared spore suspension of different test microorganisms (0.5 ml of about 106 cells/ml) was mixed with 9.5 ml of nutrient agar medium (for bacteria) at 45 °C, poured on sterile Petri dishes, and left to solidify at room temperature. Regular wells were made in the inoculated agar plates by a sterile cork borer of 0.7 mm diameter. Each well was filled with 20 mg of each tested powder. Three replicas were made for each test, and all plates were incubated at 32 °C for 24 h for bacteria. Then the average diameters of inhibition zones were recorded in centimeters and compared for all plates. MIC (minimal inhibitory concentration) was measured as follows: Half-fold serial dilutions were made for selected compounds to prepare concentrations of 10–70 mg/ ml in distilled water and zero concentration was considered as a negative control. A previously prepared pure spore suspension of each test microorganism (0.5 ml of about 106 cells/ml) was mixed with 9.5 ml of each concentration in sterile test tubes, incubated at 32 °C for 24 h for bacteria, then optical density of growth was measured by spectrophotometer (Optima SP-300, Japan) at 620 nm for each incubated mixture, results were represented graphically, and MIC was recorded for each tested material[2].

MBC and MIC for *Escherichia coli, Pseudomonas aeruginosa, Staphylococcus aureus,* and *Enterococcus faecalis* were performed in tubes by double diluting at higher dilutions. The incubation periods were examined at 37°C for 18 to 24 h in broths containing gentamicin concentrations, this was used to determine bacterial growth inhibitions. The tube for MIC exhibited growth and became turbid after incubation while the tube for MBC was still evident.

1. Shotwell, O.L., et al., *Antibiotics against plant disease. III. Duramycin, a new antibiotic from Streptomyces cinnamomeus forma azacoluta2.* 1958. **80**(15): p. 3912-3915.

2. Shadomy, S.J.M.o.c.m., *Laboratory studies with antifungal agents: susceptibility tests and bioassays.* 1985: p. 991-999.
